# Chloroplast genome assembly of *Serjania erecta* Raldk: comparative analysis reveals gene number variation and selection in protein-coding plastid genes of Sapindaceae

**DOI:** 10.3389/fpls.2023.1258794

**Published:** 2023-09-26

**Authors:** Leonardo C. J. Corvalán, Mariane B. Sobreiro, Larissa R. Carvalho, Renata O. Dias, Ramilla S. Braga-Ferreira, Cintia P. Targueta, Carlos M. e Silva-Neto, Bianca W. Berton, Ana Maria S. Pereira, José A. F. Diniz-filho, Mariana P. C. Telles, Rhewter Nunes

**Affiliations:** ^1^ Laboratório de Genética & Biodiversidade, Universidade Federal de Goiás, Goiânia, Brazil; ^2^ Instituto de Ciências Exatas e Naturais, Universidade Federal de Rondonópolis, Rondonópolis, Brazil; ^3^ Instituto Federal de Goiás, Goiás, Brazil; ^4^ Universidade de Ribeirão Preto, Ribeirão Preto, Brazil; ^5^ Laboratório de Ecologia Teórica e Síntese, Universidade Federal de Goiás, Goiânia, Brazil; ^6^ Escola de Ciências Médicas e da Vida, Pontifícia Universidade Católica de Goiás, Goiânia, Brazil

**Keywords:** cpDNA, molecular evolution, negative selection, organellar genome, plastome

## Abstract

*Serjania erecta* Raldk is an essential genetic resource due to its anti-inflammatory, gastric protection, and anti-Alzheimer properties. However, the genetic and evolutionary aspects of the species remain poorly known. Here, we sequenced and assembled the complete chloroplast genome of *S. erecta* and used it in a comparative analysis within the Sapindaceae family. *S. erecta* has a chloroplast genome (cpDNA) of 159,297 bp, divided into a Large Single Copy region (LSC) of 84,556 bp and a Small Single Copy region (SSC) of 18,057 bp that are surrounded by two Inverted Repeat regions (IRa and IRb) of 28,342 bp. Among the 12 species used in the comparative analysis, *S. erecta* has the fewest long and microsatellite repeats. The genome structure of Sapindaceae species is relatively conserved; the number of genes varies from 128 to 132 genes, and this variation is associated with three main factors: (1) Expansion and retraction events in the size of the IRs, resulting in variations in the number of *rpl22*, *rps19*, and *rps3* genes; (2) Pseudogenization of the *rps2* gene; and (3) Loss or duplication of genes encoding tRNAs, associated with the duplication of *trnH-GUG* in *X. sorbifolium* and the absence of *trnT-CGU* in the Dodonaeoideae subfamily. We identified 10 and 11 mutational hotspots for Sapindaceae and Sapindoideae, respectively, and identified six highly diverse regions (*tRNA-Lys — rps16, ndhC – tRNA-Val, petA – psbJ, ndhF, rpl32 – ccsA*, and *ycf1*) are found in both groups, which show potential for the development of DNA barcode markers for molecular taxonomic identification of *Serjania*. We identified that the *psaI* gene evolves under neutrality in Sapindaceae, while all other chloroplast genes are under strong negative selection. However, local positive selection exists in the *ndhF*, *rpoC2*, *ycf1*, and *ycf2* genes. The genes *ndhF* and *ycf1* also present high nucleotide diversity and local positive selection, demonstrating significant potential as markers. Our findings include providing the first chloroplast genome of a member of the Paullinieae tribe. Furthermore, we identified patterns in variations in the number of genes and selection in genes possibly associated with the family’s evolutionary history.

## 1 Introduction

Chloroplasts are organelles that play an essential role in photosynthesis, which, due to their origin by endosymbiosis between cyanobacteria and primitive eukaryotic cells, have their own DNA, named cpDNA or chloroplast genome (Margulis and Bermudes, 1985; Rodríguez-Ezpeleta et al., 2005; Sato, 2020). In spermatophytes, a quadripartite circular genome pattern is generally observed with two Inverted Repeat regions (IRa and IRb) separated by a Small Single Copy region (SSC) and a Large Single Copy region (LSC) (Jansen and Ruhlman, 2012; Xiao-Ming et al., 2017). Chloroplast genomes are generally used to investigate the evolutionary history between species, evaluate patterns of genetic diversity and demographic history of populations, develop DNA barcode markers for molecular taxonomic identification, and as targets for genetic transformation and production of organisms genetically modified (OGMs) (Leebens-Mack et al., 2005; CBOL Plant Working Group, 2009; Moore et al., 2010; Kang et al., 2021).

In land plants, the number of genes usually varies from 100 to 140, and in some families, such as Sapindaceae, Poaceae, Leguminosae, and Cactaceae, IR expansion and retraction events significantly influence the total number of genes (Wang et al., 2017; Xiao-Ming et al., 2017; Souza et al., 2019; Köhler et al., 2020; Dong et al., 2021). Although there is some variation in the number, the gene order generally seems to have a phylogenetic pattern of conservation in Angiosperms, showing potential to be used in comparative studies to test evolutionary hypotheses related to collinearity and synteny of plastid genes (Wicke et al., 2011). The chloroplast genome contains protein-coding and functional RNA-coding genes such as tRNAs (*trn*) and rRNAs (*rrn*). Most of the protein-coding genes are associated with cell replication mechanisms, such as the genes encoding the large subunit of ribosomal proteins (*rpl*) and the small subunit of ribosomal proteins (*rps*), photosystem proteins such as *psa* and *psb* genes, and the genes encoding NADH dehydrogenase proteins (ndh) (Allen et al., 2011; Jansen and Ruhlman, 2012).

The Sapindaceae family, also known as the soapberry family, comprises about 1900 species of 144 genera, with approximately 80% of its biodiversity contained in tropical and subtropical regions of the Southern Hemisphere (Acevedo-Rodríguez et al., 2010; Muellner-Riehl et al., 2016; Acevedo-Rodríguez et al., 2017; Buerki et al., 2021). Sapindaceae includes plants of different habits, such as trees, shrubs, lianas (woody climbing plants), and herbaceous climbing plants. It also presents species of high economic importance due to their timber, fruit, and medicinal uses (Ferrucci and Acevedo-Rodríguez, 2005; Buerki et al., 2009; Acevedo-Rodríguez et al., 2010; Muellner-Riehl et al., 2016; Dong et al., 2021). More than 50% of the tribes of the Sapindaceae family do not have a representative with a sequenced chloroplast genome, and only 17 genera contain at least one sequenced chloroplast genome (National Center for Biotechnology Information, July 7th, 2023).

The Sapindaceae family emerged at the beginning of the Upper Cretaceous (105 My) and began diversifying approximately 87 My ago in Eurasia. It later diversified throughout the Southern Hemisphere during the Paleocene. Nowadays, this family is subdivided into four subfamilies: (1) Xanthoceroideae, endemic to China; (2) Hippocastanoideae, predominantly occurring in temperate regions; (3) Dodonaeoideae, distributed in tropical and subtropical regions; and (4) Sapindoideae, with wide distribution in tropical and subtropical regions, and it is the most diverse among the four subfamilies (Buerki et al., 2009). The clades that form tribes in this family are still debated. The most recent botanical revision used proposes 20 tribes, of which 16 belong to the subfamily Sapindoideae, two tribes to the subfamily Dodonaeoideae, two tribes to the subfamily Hippocastanoideae, and in the subfamily Xanthoceratoideae no tribes were classified (Buerki et al., 2021). Although molecular studies of the group have been carried out using classical markers and next-generation sequencing, evolutionary perspectives of chloroplast genomes across tribes have yet to be evaluated.

In Sapindaceae, the genus *Serjania* Mill. has approximately 230 species distributed from the United States to Argentina. The phylogenetic relationships between species of the genus need to be better defined, presenting extensive polytomies. *Serjania* is the unique liana genus in the Sapindaceae family, and other genera of Sapindaceae are trees, shrubs, or herbs. Furthermore, it is considered the most diverse liana genus in the tropics (Acevedo-Rodriguez, 1990; Acevedo-Rodríguez et al., 2017; Buerki et al., 2021). All *Serjania* species with known karyotypes are diploid (2n=24) (Urdampilleta et al., 2012), and their genomic data are scarce, with no nuclear draft genomes or organellar genomes available in public databases. In our work, *Serjania erecta* Raldk (subfamily Sapindoideae; tribe Paullinieae) is the first liana species to have its chloroplast genome assembled and the second species of the Sapindaceae family from the Americas (the first one is *Dodonaea viscosa*) (Saina et al., 2018). *Serjania erecta* is a plant traditionally used to treat ulcers and hypertension with potential use in the treatment of Alzheimer’s disease, gastric diseases, and anti-inflammatory use (Gomig et al., 2008; Buerki et al., 2009; Hiruma-Lima et al., 2009).

Here, we assembled the chloroplast genome of *Serjania erecta*, the first one of the Paullinieae tribe (Sapindaceae), and used it in comparative analysis with species of the Sapindaceae family to elucidate evolutionary aspects that occurred in these genomes. The comparative analysis had as its main aims to identify: (1) Variations in the number of genes in plastomes of the family Sapindaceae; (2) Selection pressure on genes in chloroplast genomes of the Sapindaceae family; and (3) Regions with potential use as markers for the genus *Serjania*.

## 2 Materials and methods

### 2.1 DNA extraction and sequencing

We collected leaves from an individual of *Serjania erecta* in the Ecocerrado Brasil Private Heritage Reserve in Araxá, Minas Gerais, Brazil (19°36’47.1” S 47°08’20.9” W altitude 939 m) for DNA extraction. The species was determined using the botanical identification key for the genus (Somner et al., 2015). Plant material was identified by Dr. Inês Cordeiro (Instituto Botânico, São Paulo, São Paulo, Brazil), and a voucher specimen was deposited in the Herbarium of Medicinal Plants at UNAERP with voucher number HPMU-835.

Total DNA was extracted using the CTAB protocol (Doyle and Doyle, 1987) and quantified using horizontal agarose gel electrophoresis (1%). The library for sequencing was constructed using the SureSelectQXT kit (catalog number 5500-0120, Agilent Technologies), and the library quality validation was performed using the Bioanalyzer 2100 (Agilent). Subsequently, the library was sequenced on the MiSeq platform (Illumina) in paired-end mode (2x300) using the MiSeq v3 600 cycles kit (Illumina). All the wet laboratory steps were conducted at the Laboratory of Genetics & Biodiversity – LGBio, at the Federal University of Goiás, in Goiânia (GO) – Brazil.

### 2.2 Chloroplast genome assembly and annotation

Assembly of the *S. erecta* genome was carried out using the *Serjania polyphylla rbcL* gene (NCBI accession: GU935455.1) as a seed and 8,814,456 paired-end Illumina reads in the NOVOPlasty v3.2 program (Dierckxsens et al., 2017). *Serjania erecta* cpDNA annotation was conducted using the GeSeq webserver software (Tillich et al., 2017). In this program, the prediction of protein-coding genes and ribosomal RNAs (rRNAs) was performed using the HMMER profile Search (Wheeler and Eddy, 2013) and BLAT search (Kent, 2002) programs, and the prediction of transporter RNAs (tRNAs) using ARAGORN v1.2.38 (Laslett and Canback, 2004). Next, annotation problems were manually cured based on annotating other chloroplast genomes from the NCBI Genome as references using the Ugene v44.0 program (Okonechnikov et al., 2012). The graphical map of the chloroplast genome was made using Organelle Genome Draw (OGDRAW) version 1.3.1 (Lohse et al., 2013). The frequency of each codon was identified using the Sequence Manipulation Suite webserver: codon usage (https://www.bioinformatics.org/sms2/codon_usage).

### 2.3 Comparative genomic analysis among Sapindaceae cpDNAs

The chloroplast genome of *S. erecta* was compared to 11 species belonging to the Sapindaceae family (**Table 1**). These genomes were obtained from the Genome database (RefSeq) of the National Center for Biotechnology Information (NCBI) and represent all Sapindaceae genera deposited in this database (NCBI accession numbers in **Supplementary Table S1**, accessed on July 7, 2023). Subsequently, all genomes were annotated using the same protocol for *S. erecta*.

**Table 1:** Description of twelve chloroplast genomes of the family Sapindaceae in a comparative genomic analysis.

Species	Total length	LSC	IR	SSC	GC (%)	Protein	tRNA	rRNA	Total genes
*Serjania erecta*	159,297	84,556	28,342	18,057	37.90	87	37	8	132
*Acer buergerianum*	156,477	86,246	26,080	18,071	37.88	83	37	8	128
*Aesculus wangii*	155,871	84,882	26,390	18,210	37.95	83	37	8	128
*Dimocarpus longan*	160,833	85,709	28,427	18,270	37.79	87	37	8	132
*Dipteronia dyeriana*	157,071	85,530	26,723	18,095	37.97	85	37	8	130
*Dodonaea viscosa*	159,375	87,205	27,099	17,972	37.86	85	36	8	129
*Eurycorymbus cavaleriei*	158,777	86,941	26,922	17,992	37.92	85	36	8	129
*Koelreuteria paniculata*	163,258	90,237	27,376	18,269	37.30	85	37	8	130
*Litchi chinensis*	162,524	85,751	30,102	16,569	37.80	87	37	8	132
*Pometia tomentosa*	160,818	85,667	28,395	18,361	37.87	87	37	8	132
*Sapindus mukorossi*	160,481	85,650	27,979	18,873	37.66	87	37	8	132
*Xanthoceras sorbifolium*	161,231	85,300	28,619	18,693	37.69	86	38	8	132
Average	159,667.75	86,139.50	27,704.50	18,119.33	37.8	85.58	36.92	8	132.92
Standard deviation (SD)	2,319.68	1,493.70	1,147.41	560.61	0.18	1.51	0.51	0	1.88
Coefficient of variation (CV)	0.01	0.02	0.04	0.03	0.00	0.02	0.01	0.00	0.01

GS, chloroplast genome size; LSC, Large Single Copy region size; IR, Inverted Repeat regions size; SSC, Small Single Copy region size; GC (%), Guanine + Cytosine percentage.

To identify the size of each region of the chloroplast genomes (Small Single Copy, Large Single Copy, and Inverted Repeats) and the genes located at their ends, Geneious Prime v. 2022.2.2 was used (Kearse et al., 2012). Identifying possible rearrangement and inversion events in chloroplast genomes was performed using the Mauve v. 2.4.0 program (Darling et al., 2004).

Identification of large genomic repeat structures (forward, reverse, palindromic, and complement) was conducted in the REPuter online version program (Kurtz et al., 2001), setting the minimum repeat size to 30 bp and a Hamming distance of 3 bp. Microsatellite regions (SSR) were identified using the MISA v2.1 web tool (Beier et al., 2017). For this, the minimum repeat numbers 10, 5, 4, 3, 3, and 3 were defined for the mononucleotide, dinucleotide, trinucleotide, tetranucleotide, pentanucleotide, and hexanucleotide repeats, respectively.

### 2.4 Phylogenetic analysis

We performed three phylogenetic reconstructions: One for the Sapindaceae family to understand the phylogenetic relationship of *S. erecta* with other chloroplast genomes of the family; the other two phylogenies aimed to test whether the available molecular markers of chloroplast genomes together with the currently available *S. erecta* data are capable of clarifying the phylogenetic relationships of the genus *Serjania*.

For the phylogenetic reconstruction of the chloroplast genomes of the family Sapindaceae, 23 species were selected (**Supplementary Table S1**), of which 22 species belonged to the order Sapindales and one species of the order Malvales (*Gossypium hirsutum*), used as an outgroup (external group) in rooting from the tree. Among the species of the order Sapindales, 16 belong to the Sapindaceae family (*Serjania erecta*, *Acer buergerianum*, *Acer truncatum*, *Acer longipes*, *Aesculus chinensis*, *Aesculus wangii*, *Dimocarpus longan*, *Dipteronia sinensis*, *Dipteronia dyeriana*, *Dodonaea viscosa*, *Eurycorymbus cavaleriei*, *Koelreuteria paniculata*, *Litchi chinensis*, *Pometia tomentosa*, *Sapindus mukorossi*, and *Xanthoceras sorbifolium*), two species belonging to the Meliaceae family (*Khaya senegalensis* and *Cedrela odorata*), two species of the Rutaceae family (*Citrus sinensis* and *Ruta graveolens*). *Mangifera indica* and *Boswellia sacra* were selected for the Anacardiaceae and Burseraceae families, respectively. The CDS of these species were obtained from GenBank. Seventy-four orthologous CDS were analyzed, retaining only one copy for each duplicated gene and excluding the *rpl22*, *rps2*, *rps11*, and *rps19* genes.

The CDS were aligned using MAFFT v.7 (Katoh and Standley, 2013) and concatenated using Sequence Matrix v.1.7.8 (Vaidya et al., 2011). The most informative regions for molecular phylogeny were selected using the Gblocks webserver (Talavera and Castresana, 2007). Phylogenetic reconstruction was performed using the maximum likelihood (ML) method in the IQ-TREE v.1.6.12 program (Nguyen et al., 2015). In the construction of the phylogenetic tree, 63979 bp were used, and the best model was selected using the ModelFinder (Kalyaanamoorthy et al., 2017) implemented in the IQ-TREE (Nguyen et al., 2015). The GTR+F+R3 model was selected, and the tree node support values were evaluated using the bootstrapping method with 1000 replicates.

The other two phylogenetic trees for the genus *Serjania* were constructed based on previously published marker data (Buerki et al., 2009; Buerki et al., 2010; Acevedo-Rodríguez et al., 2017). These two phylogenies were predicted using the following data: the first using data from the *matK*, *rpoB*, *trnD-trnT*, *trnK-matK*, *trnL*(intron), and *trnL-trnF* markers and the second other information only from *trnL* (intron) (**Supplementary Table S2**). For *S. erecta*, such regions were extracted from the chloroplast genome. In these phylogenies, we followed the same pipeline used in the ML tree of the CDS of chloroplast genomes. The best evolutionary model of nucleotide substitution for both trees was K3Pu+F.

### 2.5 Nucleotide diversity and molecular evolution in Sapindaceae cpDNAs

In the analysis of nucleotide diversity, the same twelve species used in the comparative analyzes were used (**Table 1**), separating them into two groups: (1) formed by the 12 species of the Sapindaceae family and (2) a subgroup of 6 species belonging to the Sapindoideae subfamily. The genomes of the two groups were aligned using the MAFFT program (Katoh and Standley, 2013), and subsequently, the calculation of nucleotide diversity (π) was performed using the DnaSP v6 program defining windows of 600 bp and steps of 200 bp, (Rozas et al., 2017).

To understand the process of evolution of chloroplast genes, we calculated the ratios between non-synonymous and synonymous mutations (ka/ks). When higher than 1.0, this ratio suggests a positive selection process; when less than 1, it suggests a negative selection process; when equal to 1.0, it indicates the absence of selection (Nei and Kumar, 2000). The calculation was performed using the 77 CDS in common between the chloroplast genomes of the 12 species of Sapindaceae aligned in the MAFFT program (Katoh and Standley, 2013). The value of ka/ks was estimated considering two neutral models, one considering a phylogenetic tree (runmode = 0; model = 0; NSsites = 0) and the other only the pairwise relationship (runmode = -2; model = 0; NSsites = 0) and establishing a minimum number of four synonymous mutations, through the PAML v.4.9 program (Yang, 2007).

The presence of local positive selection (ka/ks > 1) in all 77 genes was investigated, using the contrast between selection models and neutral models: (1) M2a (positive selection model) x M1a (model neutral); (2) M8 (positive selection model with beta distribution) x M7 (neutral model with beta distribution); (3) M8 (positive selection model with beta distribution) x M8a (null hypothesis model). To test the significance of the contracts, we calculated Likelihood Ratio Tests (LRT) and the False Discovery Rate (FDR), both in R language (R Core Team, 2020).

## 3 Results

### 3.1 The chloroplast genome of *Serjania erecta*

**Figure 1:**
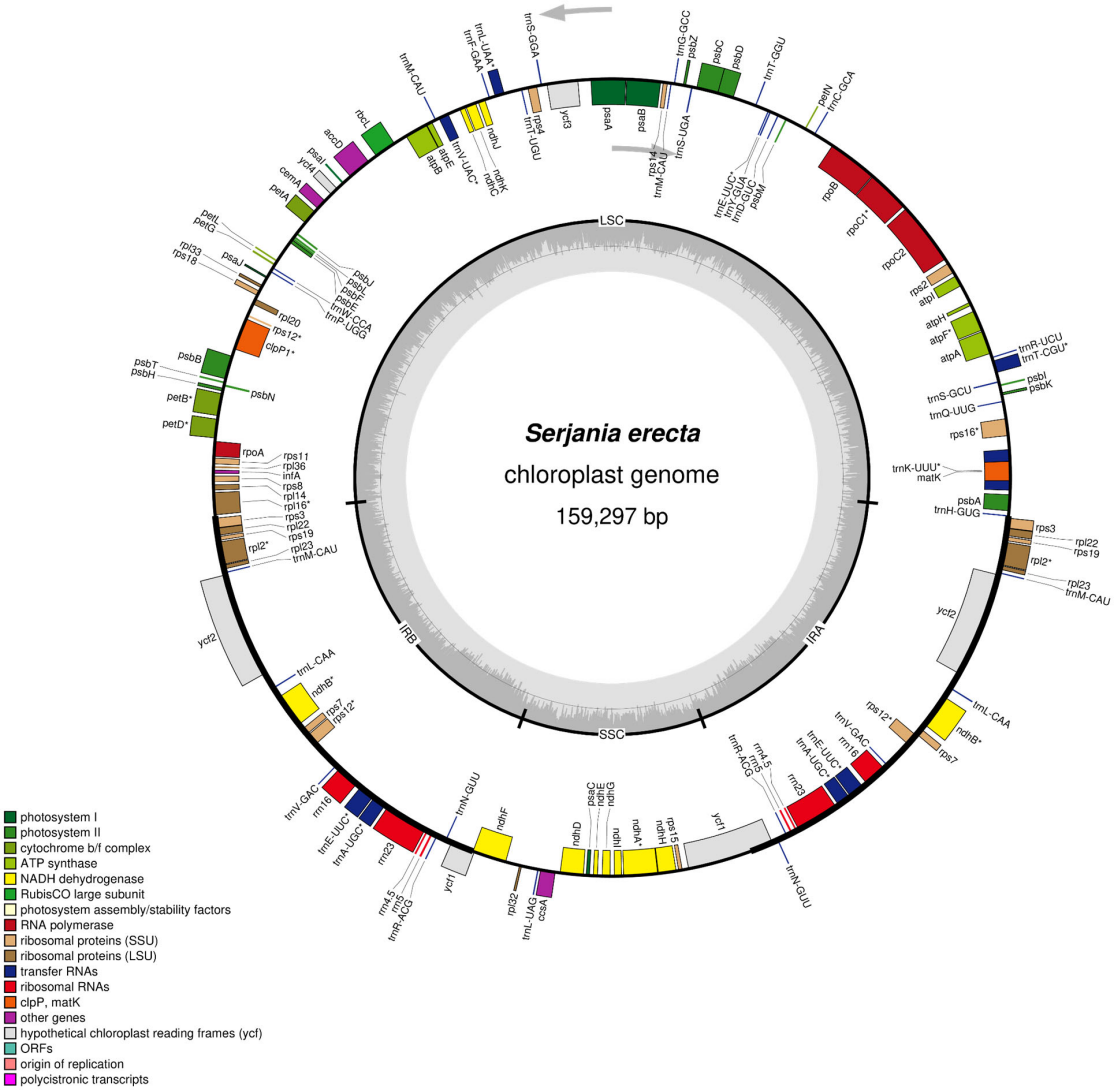
Chloroplast genomic map of *Serjania erecta* Radlk (Sapindaceae: Sapindoideae). The genes colored according to the functional group are represented in the outermost circle, the genes external to the circumference are transcribed in a counterclockwise direction, and the internal genes are transcribed in a clockwise direction (the gray arrows indicate the beginning). The inner circle represents Inverted Repeat regions (IRa and IRb), Large Single Copy (LSC), and Small Single Copy (SSC) regions. The bar graph corresponds to the GC content. The asterisk highlights genes with introns.

We generated 8.8 million paired-end reads to assemble the complete chloroplast genome sequence of *S. erecta*. This species has a chloroplast genome size of 159,297 bp (871x of average coverage) and a guanine + cytosine content (GC%) of 37.9% (**Figure 1** and **Table 1**). Furthermore, the *S. erecta* cpDNA has the typical quadripartite structure of Embryophyte chloroplasts with a Large Single Copy (LSC) region of 84,556 bp, a Small Single Copy (SSC) region of 18,057 bp, and two Inverted Repeat regions (IRa and IRb) of 28,342 bp separating the SSC and LSC regions (**Figure 1** and **Table 1**).

We annotated 132 genes and two pseudogenes (*infA* and *ycf1*) in the chloroplast genome of *S. erecta*, 87 protein-coding genes, and 45 functional RNAs (**Table 1** and **Supplementary Table S3**). Among the protein-coding genes, nine genes are duplicated in the IR regions (*rpl2*, *rpl22*, *rpl23*, *rps3*, *rps7*, *rps12*, *rps19*, *ndhB*, and *ycf2*), nine genes have an intron (*atpF*, *rpl2*, *rps12*, *rpl16*, *rps16*, *rpoC1*, *ndhA*, *ndhB*, *petB*, and *petD*) and three genes have two introns (*clpP*, and *ycf3*) (**Supplementary Table S1**). We also identified 37 tRNA genes and eight rRNA genes. Considering the tRNA genes, *trnM-CAU* has four copies, and *trnA-UGC*, *trnE-UUC*, *trnL-CAA*, *trnN-GUU*, *trnR-ACG*, and *trnV-GAC* have two copies each. Five tRNA genes contain an intron (*trnA-UGC*, *trnK-UUU*, *trnL-UAA*, *trnT-CGU*, and *trnV-UAC*). There are four types of rRNA genes in the *S. erecta* cpDNA (*rrn4.5*, *rrn5*, *rrn16*, and *rrn23*), and they are all located in the IR regions and are therefore duplicated (**Supplementary Table S3**).

We identified 26,991 codons in the *S. erecta* chloroplast genome (**Supplementary Table S4**). The most used were AAA (Lys) and ATT (Ile), representing 1,136 (4.20%) and 1,131 (4.19%) of the total codons, respectively. Among amino acids, the most frequent is leucine (Leu), with 2,835 amino acids (10.32% of total amino acids), followed by isoleucine (Ile) and Serine (Ser), with 2,280 amino acids (8.45% of total amino acids) and 2,068 amino acids (7.66% of total amino acids), respectively.

### 3.2 Sapindaceae chloroplastidial genome structure

We performed a comparative analysis with the chloroplast genomes of *S. erecta* and 11 other cpDNAs from species of the Sapindaceae family. The chloroplast genome of *S. erecta* has a structure and genomic size similar to that of other Sapindaceae species (**Table 1**), standing out only as the species with the smallest LSC size, with 84,556 bp, which ranged up to a maximum length of 90,237 bp (*Koelreuteria paniculata*). The total size of chloroplast genomes ranged from 163,258 bp (*K. paniculata*) to 155,871 bp (*Aesculus wangii*). The mean GC content among the twelve Sapindaceae is 37.80%, with a standard deviation of 0.18 (**Table 1**).

**Figure 2:**
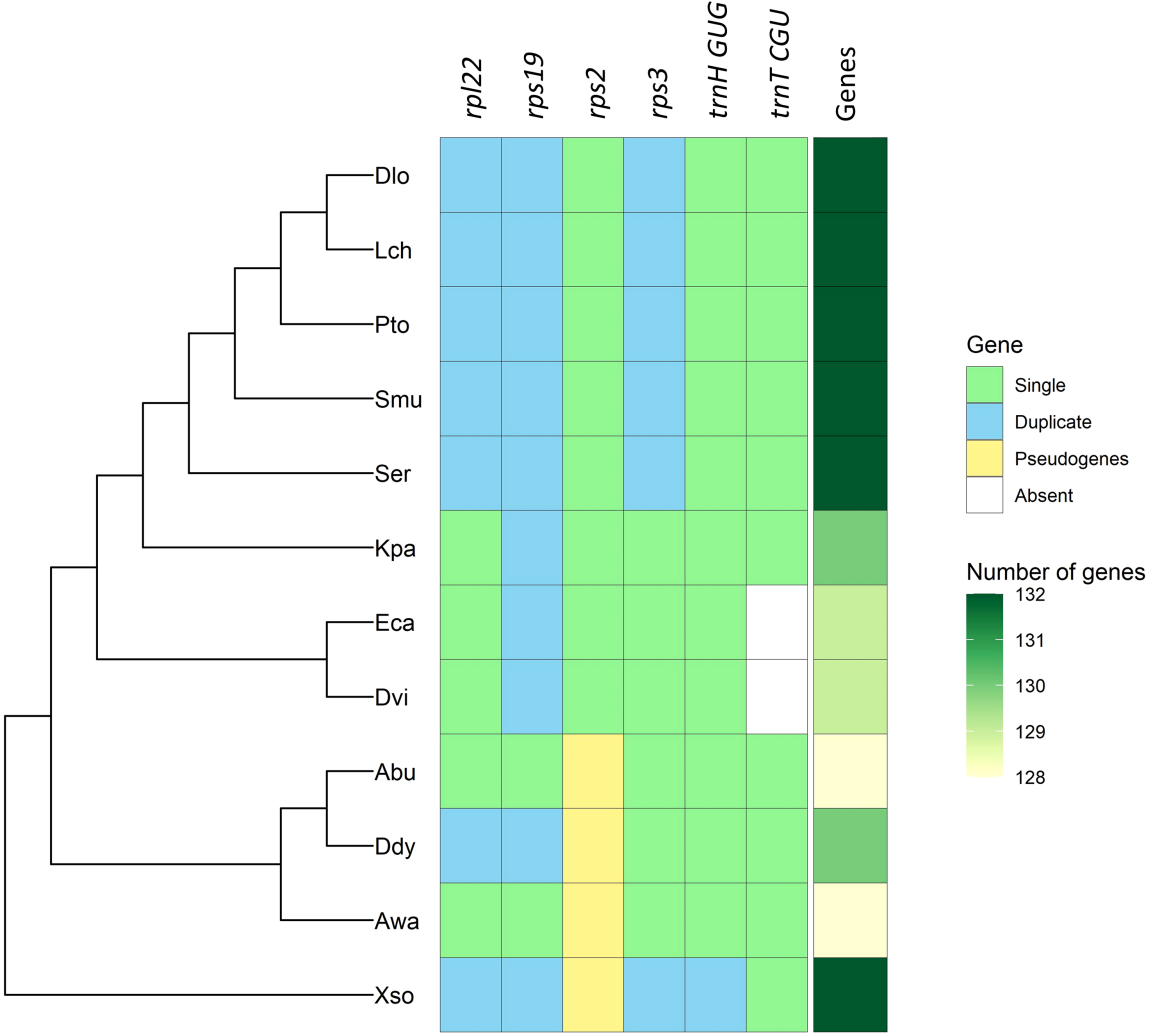
Variation in the number of genes in chloroplast genomes of the Sapindaceae family ordered by phylogeny. *Acer buergerianum* (Abu), *Aesculus wangii* (Awa), *Dimocarpus longan* (Dlo), *Dipter dyeoniariana* (Ddy), *Dodonaea viscosa* (Dvi), *Eurycorymbus cavaleriei* (Eca), *Koelreuteria paniculata* (Kpa), *Litchi chinensis* (Lch), *Pometia tomentosa* (Pto), *Sapindus mukorossi* (Smu), *Serjania erecta* (Ser), and *Xanthoceras sorbifolium* (Xso).

The total number of genes in the chloroplast genomes of the family Sapindaceae ranges from 128 to 132. The most significant number of genes is described in five of the six species belonging to the subfamily Sapindoideae (*Dimocarpus longan*, *Litchi chinensis*, *Pometia tomentosa*, *Sapindus mukorossi*, and *S. erecta*) and in the species *Xanthoceras sorbifolium* (subfamily Xanthoceratoideae). The smallest number of genes was observed in the *Acer buergerianum* and *A. wangii*, both species belonging to the subfamily Hippocastanoideae (**Figure 2**). Chloroplast genomes with fewer genes also had smaller IR sizes (**Table 1**), which ranged from 26,080 bp in *A. buergerianum* species to 30,102 bp in *Litchi chinensis*.

All Sapindaceae species analyzed showed the same four rRNA genes (*rrn4.5*, *rrn5*, *rrn16*, and *rrn23*) duplicated in the IRa and IRb regions. The tRNA genes ranged from 38 genes in *X. sorbifolium* to 36 genes identified in the two species of the subfamily Dodonaeoideae (*Dodonaea viscosa* and *Eurycorymbus cavaleriei*). Furthermore, it is possible to observe specific genomic patterns in the analyzed genomes, such as the duplication of the *trnH-GUG* gene in the *X. sorbifolium* genome and the deletion of the *trnT-CGU* gene in the Dodonaeoideae subfamily (**Figure 2**). There were no differences regarding the number of introns in the genes of the 12 species of the Sapindaceae family (**Supplementary Table S5**).

**Figure 3:**
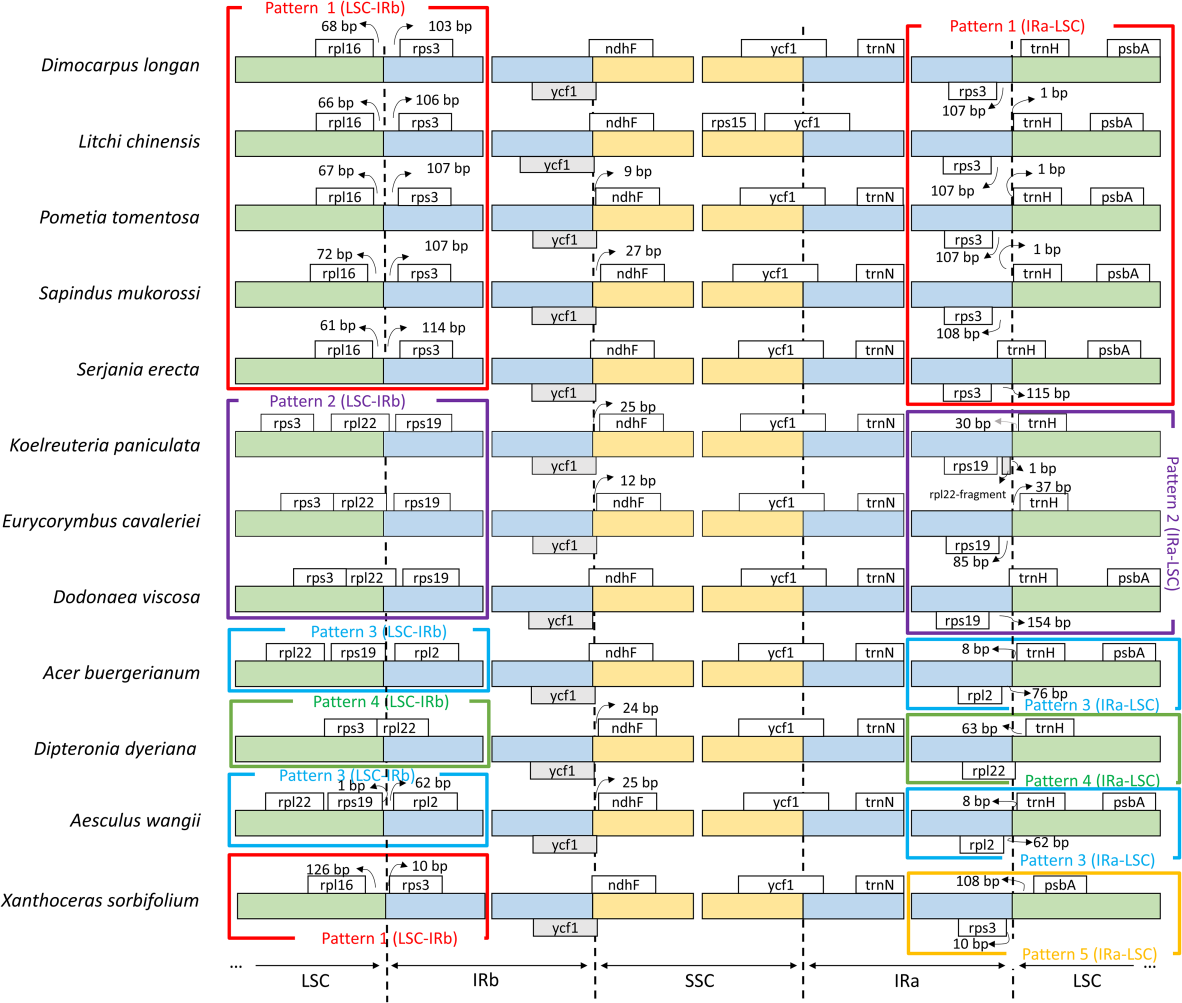
Comparison between the borders of the regions that delimit the chloroplast genomes of species from the Sapindaceae family. Inverted repeat regions (IRa and IRb) are represented in blue, the large single-copy region (LSC) in green, and the small single-copy region (SSC) in yellow. The white boxes above the bars represent counterclockwise transcribed genes, and those below represent clockwise transcribed genes. The gray boxes represent pseudogenes.

The genes that flank the borders of the chloroplast genome regions were identified to observe patterns related to the presence and position of genes (**Figure 3**). Four distinct patterns appear in the border region between LSC and IRb: i) Patterns 1 - LSC-IRb flanked by the rpl16 and rps3 genes, in the species *D. longan*, *L. chinensis*, *P. tomentosa*, *S. mukorossi*, *S. erecta*, and *X. sorbifolium*; ii) Patterns 2 - LSC-IRb flanked by the *rp122* and *rps19* genes, was identified in the species *D. viscosa*, *E. cavaleriei*, and *K. paniculatai*; iii) Patterns 3 - LSC-IRb flanked by the *rps19* and *rpl2* genes, was identified in *A. buergerianum* and *A. wangii*; and iv)Patterns 4 - LSC-IRb in a single *D. dyeriana* species, flanked by the *rps3* and *rpl22* genes.

Five distinct patterns were identified in the border region between IRa and LSC. In Standard 1, the LSC-IRa region is flanked by *rps3* and *trnH* in the species *D. longan*, *L. chinensis*, *P. tomentosa*, *S. mukorossi*, and *S. erecta*. The *X. sorbifolium* species is the only one where the IRa-LSC region is flanked by *rps3* and *psbA*, being the pattern 5 (IRa-LSC). pattern 2 (*trnH* and *rps19*), pattern 3 (*trnH* and *rpl2*), and patterns 4 (*trnH* and *rpl22*) (**Figure 3**). The same genes in all species flanked the transition regions between IR and SSC: between IRb and SSC by pseudogenized *ycf1* and *ndhF* and between SSC and IRa by the *ycf1* gene (**Figure 3**).

We identified only one chloroplast genome rearrangement event for the twelve Sapindaceae species analyzed, which resulted in the unique genomic organization of the cpDNA of *K. paniculata* (**Supplementary Figure S1**). In total, we identified three syntenic blocks that are divided due to a region located in the IRa of *K. paniculata*, which is in the reverse complementary orientation compared to the other species (block in green, **Supplementary Figure S1**).

### 3.3 Repetitive portions of the chloroplastidial genome of Sapindaceae

**Figure 4:**
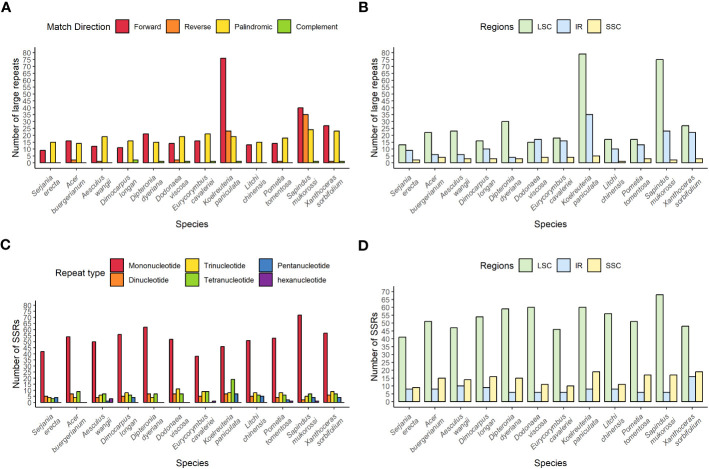
Distribution of repeats in the chloroplast genomes of twelve species of the Sapindaceae family. **(A)** Distribution of large repeats by type. **(B)** Distribution of large repeats according to their region in the chloroplast genome. **(C)** Distribution of SSRs according to repetition motif. **(D)** Distribution of SSRs according to their region in the chloroplast genome.

The number of large repeats among the 12 species in the comparative analysis ranged from 24 in S. erecta to 119 in *K. aniculate* (**Figure 4A**). In the Sapindaceae family, it was possible to find the four types of large repeats (forward, reverse, complement, and palindromic) in only four species, and the most frequent types were forward and palindromic. The large repeats are generally found in the LSC regions, followed by the IR, and less often in the SSC region in all species but *D. viscosa* (**Figure 4B**).

*S. erecta* has the lowest number of SSRs, and *Sapindus mukorossi* has the highest number among all the analyzed Sapindaceae species, with 58 and 91 SSR repeats, respectively (**Figure 4C**). Mononucleotide SSR repeats were the most abundant in all species. The SSRs are mainly located in the LSC and less frequently in the IR in all species (**Figure 4D**).

### 3.4 Molecular evolution in chloroplast genomes of Sapindaceae

**Figure 5:**
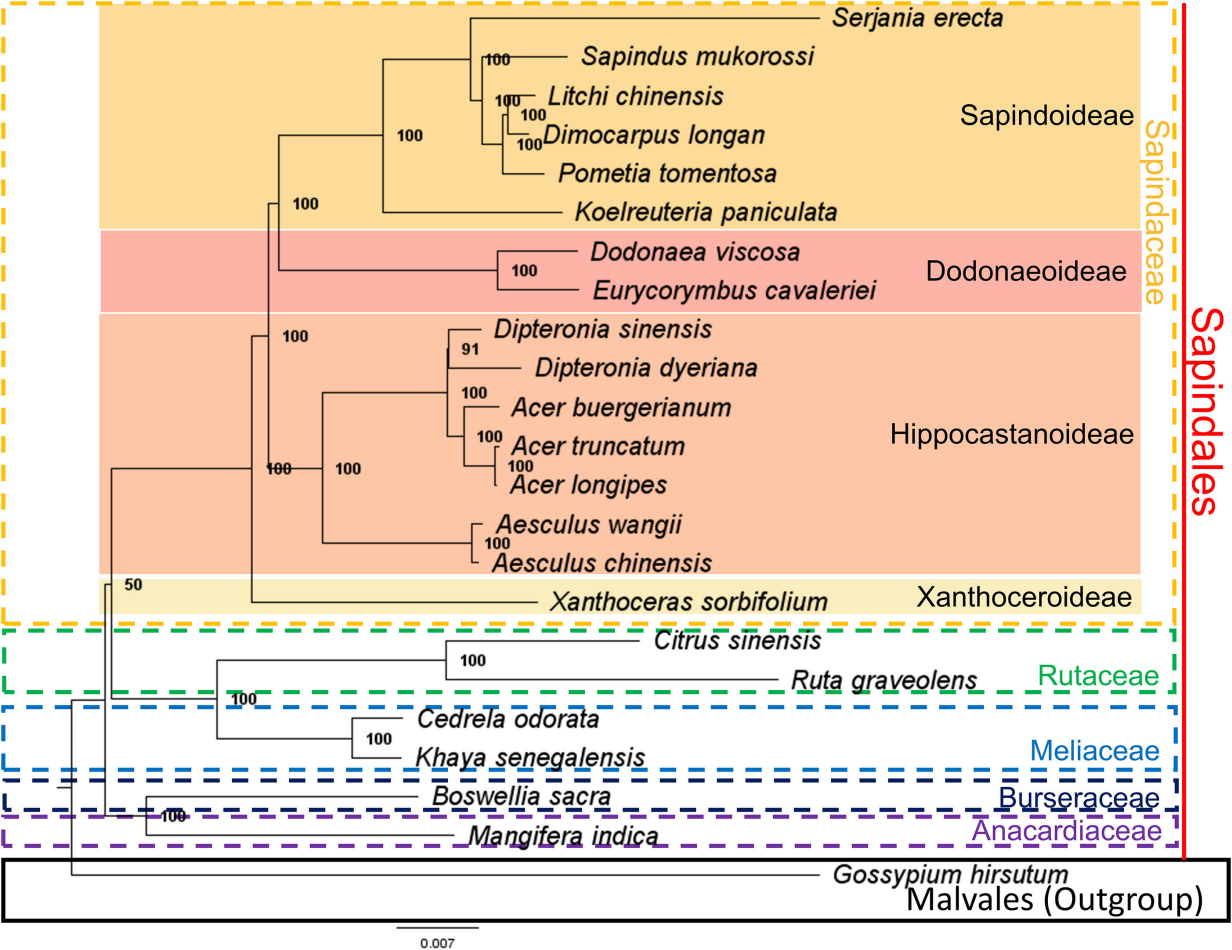
The phylogenetic tree of the Sapindaceae family was obtained by the maximum likelihood (ML) method and proposed using 74 protein-coding genes and nucleotide sequences from the chloroplast genomes of 23 species. Numbers on the nodes represent the Bootstrap values obtained from 1000 replicates. Additionally, species from Rutaceae, Meliaceae, Burseraceae, and Anacardiaceae were added. Malvales order were used as outgroups.

We reconstructed a phylogenetic tree of the Sapindaceae family using a 68,917 bp hypermatrix composed of nucleotides of 74 orthologue protein-coding genes in the chloroplast genomes of the 23 species analyzed. The phylogenetic analysis resulted in a tree with high support values for the nodes, except for the branching between the family Sapindaceae and the clade formed by the families Rutaceae and Meliaceae (bootstrap support value = 50) (**Figure 5**). In the Sapindaceae family, it was possible to identify the four monophyletic clades formed by the four subfamilies: Sapindoideae, Dodonaeoideae, Hippocastanoideae, and Xanthoceroideae, in agreement with the topologies proposed by Bueki et al. (2009); Bueki et al. (2021); Acevedo-rodríguez et al. (2017), and Angiosperm Phylogeny Group (APG) IV (Chase et al., 2016). The phylogeny for the genus *Serjania* also presented itself as a monophyletic clade, concordant with the taxonomic changes that suggested the inclusion of different genera as *Serjania*, proposed by Acevedo-Rodríguez et al. (2017) (e.g., *Balsas guerrerensis* as *Serjania guerrerensis* (Cruz Durán & K.Vega) Acev.-Rodr.; *Houssayanthus biternatus* as *Serjania biternata* (Weath.) Acev.-Rodr.; *Houssayanthus incanus* as *Serjania incana* Radlk.) (**Supplementary Figure S2**). Our phylogenetic tree is also similar to the reconstruction obtained by Steinmann et al. (2022), which was based on nuclear ITS and chloroplast trnL-F sequences (**Supplementary Figures S2A, B**).

**Figure 6:**
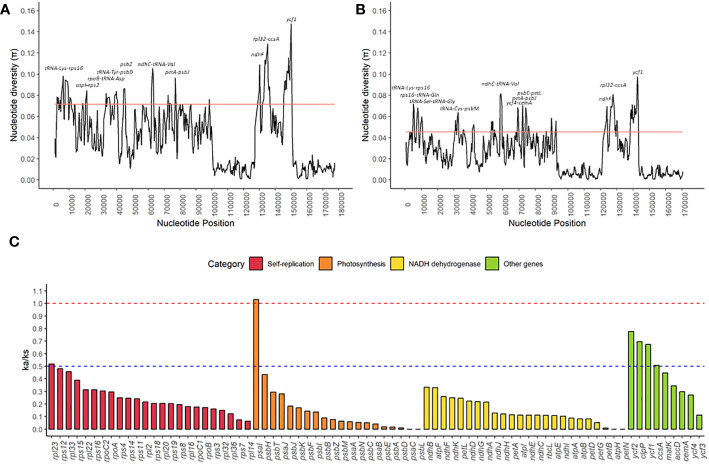
Nucleotide and mutational variation in chloroplast genomes of Sapindaceae family: **(A)** Distribution of nucleotide diversity along the chloroplast genomes of twelve species of the Sapindaceae family. **(B)** Distribution of nucleotide diversity along the chloroplast genomes of six species of the subfamily Sapindoideae. **(C)** Relation of non-synonymous mutations and synonymous mutations (ka/ks) for the 77 protein-coding genes found in all chloroplast genomes from the twelve species of the Sapindaceae family. The solid red line in graphs **(A)** and **(B)** indicates twice the chloroplast genome’s median nucleotide diversity (π) values. The dashed red line indicates the value at which the ka/ks ratio equals 1.0, and the dashed blue line indicates the value at which the ka/ks ratio equals 0.5.

The nucleotide diversity (π) ranged from 0.0008 to 0.1473 for the Sapindaceae family, with a median of 0.0357 (**Figure 6A** and **Supplementary Table S6**). For the subfamily Sapindoideae, the variation of nucleotide diversity was from 0 to 0.0974, with a median of 0.0227 (**Figure 6B** and **Supplementary Table S7**). Nucleotide diversity hotspots were defined as sites with values above twice the median. For the Sapindaceae family we identified ten nucleotide diversity hotspots (*tRNA-Lys* – *rps16*, *atpI – rps2*, *rpoB* – *tRNA-Asp*, *tRNA-Tyr* – *psbD*, *psbZ*, *ndhC* – *tRNA-Val*, *petA* – *psbJ*, *ndhF*, *rpl32 – cssA*, and *ycf1*) (**Figure 6A**), and for the Sapindoideae subfamily we identified 11 nucleotide diversity hotspots (*tRNA-Lys – rps16*, *rps16 – tRNA-Gln*, *tRNA-Ser – tRNA-Gly*, *tRNA-Cys – psbM*, *ndhC – tRNA-Val*, *ycf4 – cemA*, *petA – psbJ*, *psbE – petL*, *ndhF*, *rpl32 – cssA*, and *ycf1*) (**Figure 6B**). *tRNA-Lys — rps16*, *ndhC – tRNA-Val, petA – psbJ*, *ndhF*, *rpl32 – ccsA*, and *ycf1* are hotspots for both Sapindaceae family and Sapindoideae subfamily.

The mean values of the ratios of non-synonymous mutations and synonymous mutations (ka/ks) were 0.2160 (SD = 0.19), ranging from 0.001 for the *atpH*, *petN*, *psaC*, and *psbL* genes to 1.032 for the *psaI* gene (**Figure 6C**). We did not identify any gene with ka/ks values significantly greater than 1.0. Only the *psaI* gene showed a ratio close to 1, demonstrating that of the 77 common genes of chloroplast genomes, 76 genes are under strong negative selection.

**Figure 7:**
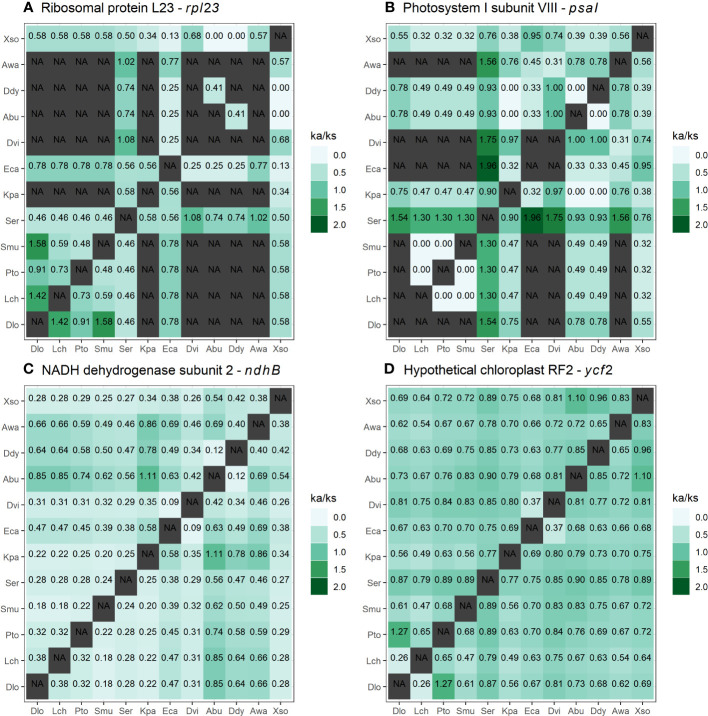
Relationships between pairwise non-synonymous and synonymous (ka/ks) mutations, considering the chloroplast protein-coding genes of 12 species of Sapindaceae. **(A)** Heatmap for the *rpl23* gene (Self-replication). **(B)** Heatmap for the *psaI* gene (photosynthesis). **(C)** Heatmap for the *ndhB* gene (NADH dehydrogenase). **(D)** Heatmap for the *ycf2* gene (other genes). Values equal to NA represent outliers, genes with fewer synonymous mutations than necessary for calculating ka/ks. *Acer buergerianum* (Abu), *Aesculus wangii* (Awa), *Dimocarpus longan* (Dlo), *Dipter dyeoniariana* (Ddy), *Dodonaea viscosa* (Dvi), *Eurycorymbus cavaleriei* (Eca), *Koelreuteria paniculata* (Kpa), *Litchi chinensis* (Lch), *Pometia tomentosa* (Pto*), Sapindus mukorossi* (Smu), *Serjania erecta* (Ser), and *Xanthoceras sorbifolium* (Xso).

To understand how natural selection works in different species in pair-by-pair comparisons, the genes with the highest ka/ks values in each of the functional groups of genes were selected (**Figure 7**). For the *rpl23* and *psaI* genes (**Figures 7A, B**), outliers (NA) were identified, that is, genes in which the ka/ks ratios did not show the defined minimum number of synonymous mutations. The *ndhB* and *ycf2* genes (**Figure 6C**) showed the highest values of ka/ks ratio for the categories of NADH dehydrogenase and “other genes”, respectively, showing punctual, positive selection relationships (**Figures 7C, D**). In particular, *S. erecta* displayed the *psaI* gene with ka/ks values greater than 1 for 7 of the 11 species that were pairwise compared (**Figure 7B**).

We identified local positive selection signals for the three tested contrasts evidenced based on LTR and FDR (M2a x M1a; (2) M8 x M7; and (3) M8 x M8a) (**Supplementary Table S8**). Among these, the contrast between the M8 x M7 models showed the highest number of genes under local positive selection with 12 genes (*atpA*, *clpP*, *matK*, *ndhA*, *ndhF*, *petD*, *psaB*, *rpl32*, *rpoB*, *rpoC2*, *ycf1*, and *ycf2*). As for the contrasts between the M2a x M1a and M8 x M8a models, we identified positive local selection for four genes (*ndhF*, *rpoC2*, *ycf1*, and *ycf2*). The *psaI* gene was the only one that showed neutral evolution (ka/ks≅1) and showed significant positive selection for the LTR test.

## 4 Discussion

The chloroplast genomes of the family Sapindaceae have a relatively conserved structure for the twelve species studied (**Table 1**). They show slight variation in genome size and GC content. Among the regions of the plastid genomes, the IRs showed the highest variation, with variation in the genes that flank the transition between the LSC and IR regions being associated with gene abundance (**Table 1**; **Figure 3**). These results suggest that IR expansion and retraction events are an essential source of variation in the number of genes in the Sapindaceae family, with a possible phylogenetic relationship. The *S. erecta* cpDNA has genomic size, structure, number of genes, and GC content within the range expected for chloroplast genomes of the family Sapindaceae (**Table 1**). The GC content value for this species is most similar to that described for *Dodonaea viscosa* and *Acer buergerianum*. Among the twelve species analyzed, the GC content was highly conserved, as described so far for the Sapindaceae family (Saina et al., 2018; Ma et al., 2019; Dong et al., 2021)

A slight variation in the size of the four regions of the chloroplast genomes (LSC, SSC, IRa, and IRb) was observed, considering the coefficient of variation (CV), the sizes of the IRs (CV=0.04) and SSC (CV=0.03) varied more than the LSC (CV=0.01), which is an expected pattern for land plants (**Table 1**). (Xiao-Ming et al., 2017). This variation is related to expansion and retraction events in the size of IRs, events already described in members of the Sapindaceae family and other angiosperms as an essential evolutionary mechanism in land plants (Dugas et al., 2015; Li et al., 2017; Saina et al., 2018; Wang et al., 2018; Ma et al., 2019; Dong et al., 2021).

The number of genes in the chloroplast genomes of the Sapindaceae family is consistent with previously published studies (Dong et al., 2021), suggesting that this variability in the number of genes is due to three factors: the expansion and retraction in the size of the IRs, the process of pseudogenization of the *rps2* gene, and the mobility of the tRNA genes (**Figure 2**). The evidence of expansion and retraction processes in the IR regions comes from the gene colinearity in the transition of the IR regions with the LSC and SSC regions (Li et al., 2017). Thus, the identified patterns provide evidence for a possible phylogenetic relationship between the IR expansion and contraction events with the number of genes. The genes *rpl22*, *rps19*, and *rps3*, located in the IR in the subfamily Sapindoideae, except for the most basal species *Koelreuteria paniculata* (**Figure 2**), are part of a set of genes encoding ribosomal proteins (Christopher et al., 1988). Among these three genes, the *rps19* gene is commonly found in the IR in dicotyledons, although in other families, such as Euphorbiaceae, this gene can be found in both the IR and LSC regions (Li et al., 2017). The *rpl22* gene, found in the chloroplast genomes of angiosperms except for the Fabaceae family, is located in the IR of the Rutaceae family (order Sapindales), indicating IR expansion events (Gantt et al., 1991; Bausher et al., 2006). Previous studies for the Sapindaceae family also showed expansion and retraction processes in the LSC-IRb region, occurring even within genera of the Sapindaceae family, highlighting that the IR expansion and retraction events are an essential source of variability in the number of genes in the Sapindaceae (Li et al., 2017; Xiao-Ming et al., 2017; Saina et al., 2018; Ma et al., 2019; Köhler et al., 2020).

The *rps2* gene produces the 2S ribosomal protein and is pseudogenized in Hippocastanoideae and Xanthoceratoideae subfamilies (Zhou et al., 2016; Chen et al., 2019). This gene is often under positive selection in other angiosperm families and is located near a nucleotide diversity hotspot (*atpI-rps2*). Therefore, the accumulation of nonsynonymous mutations may have led to the pseudogenization of this gene (Wu et al., 2018; Li et al., 2020a; Li et al., 2020b; Sobreiro et al., 2020). The *trnH-GUG* gene is located exclusively in internal regions of the IRs in *X. sorbifolium*, so this duplication may not be associated with IR expansion events (**Figure 2**). Furthermore, other variations in genes encoding tRNAs have been identified, as the absence of *trnT-CGU* genes in *Eurycorymbus cavariei* has already been described (Dong et al., 2021). The chloroplasts of *D. viscosa* show the same absence, perhaps indicating a synapomorphy of the subfamily Dodonaeoideae (Dong et al., 2021). The *trnT-CGU* gene is not the only gene encoding threonine transporter RNAs, and its absence may be supplied by the other two threonine transporter RNAs in the subfamily Dodonaeoideae.

There is a high level of gene conservation in the cpDNAs of the Sapindaceae family, all of which have the same number of introns. The *clpP*, *rps12*, and *ycf3* genes have two introns and were observed in all annotated species, unlike some annotations that did not identify the *rps12* gene (**Supplementary Table S5**). The *rps12* gene has two 3’ exons in the IR and one 5’ exon in the LSC and is a trans-spliced gene. The trans-spliced *rps12* gene has been reported in several Viridiplantae. In some taxonomic groups, such as ferns, one intron of *rps12* has been lost (Hildebrand et al., 1988; Ma et al., 2019; Sobreiro et al., 2020). We identified two pseudogenes (*infA* and one of the *ycf1* copies) in all Sapindaceae species. The *infA* gene is a mobile gene that has undergone several transfer events from the chloroplast to the nucleus, mainly in lineages of the Rosid clade (Millen et al., 2001). Pseudogenization of one of the ycf1 copies is common in angiosperm chloroplast genomes and occurs due to its location in the transition zone between the IR and SSC regions (**Figure 3**) (Li et al., 2017).

Among the species we identified repetitive regions, *K. paniculata* and *S. erecta* had the highest and lowest amounts of wide repeats, respectively. *Sapindus mukorossi* and *S. erecta* are the species with the highest and lowest amounts of microsatellite repeats (**Figure 4**), respectively, confirming a previous study highlighting that *S. mukorossi* has large numbers of repetitive regions in its chloroplast (Dong et al., 2021). Such repetitive regions are highly informative about genetic diversity within species and are commonly used as molecular markers in studies of biogeography and population genetics (Varshney et al., 2005).

Leucine and cysteine are the most and least abundant amino acids constituting the proteins of the *S. erecta* chloroplast genome (**Supplementary Table S5**), as previously described in other species of Sapindaceae and angiosperms (Muellner-Riehl et al., 2016; Ma et al., 2019). The predominant use of some amino acids in chloroplasts may be related to the conservation of genes encoding tRNAs since the composition and number of tRNAs affect the codon composition (Novoa et al., 2012).

The ka/ks ratio is a valuable analysis for understanding the evolutionary process within genes, so values greater than 1 (Ka/Ks > 1) indicate positive selection, values equal to 1 (Ka/Ks = 1) indicate neutrality and values less than 1 (Ka/Ks < 1) indicate negative selection (Nei and Kumar, 2000). We found no evidence of positive selection for any of the cpDNA protein-coding genes (ka/ks > 1) (**Figure 6C**). Only the *psaI* gene is under neutrality, indicating a high conservation of amino acid sequences produced by chloroplast protein-coding genes in Sapindales. The *psaI* gene makes up subunit VIII of the photosystem I reaction center and showed evidence of local positive selection in other studies (Plöchinger et al., 2016; Schöttler et al., 2017). Previous tests in *Nicotiana tabacum*, inducing mutations in this gene, did not cause changes in plant growth and only responded to high light and cold stress during leaf senescence (Schöttler et al., 2017). Although *psaI* is the only gene under neutral evolution, several of the ka/ks relationships did not show synonymous mutations (ka), and the highest values of the relationships are primarily associated with *S. erecta* genes (**Figure 7B**). A pairwise comparison of *S. erecta* with the other species for the *psaI* gene shows an accumulation of non-synonymous mutations in this species, indicating possible positive selection. This heterogeneous speciation process in the *psaI* gene may be associated with evolutionary factors of the tribe or species. The *S. erecta* is the unique Sapindaceae liana with data available; positive selection for *psaI* may be linked to this habit, as this gene is associated with responses to light and cold. We suggest that comparisons of this gene with orthologous genes from species closer to *S. erecta* are needed to assess the evolutionary impact of these non-synonymous mutations.

The contrasts between the M8 x M7 models identified 12 genes (*atpA*, *clpP*, *matK*, *ndhA*, *ndhF*, *petD*, *psaB*, *rpl32*, *rpoB*, *rpoC2*, *ycf1*, and *ycf2*) under local positive selection (**Supplementary Table S8**), among these genes one is an ATP subunit (*atpA*), one encodes a protease (*clpP*), one encodes maturase (*matK*), two encodes NADH dehydrogenase subunits (*ndhA* and *ndhF)*, one composes the Cytochrome b/f complex (*petD*), one encodes a Photosystem I subunit (*psaB*), one encode a ribosomal protein subunit (*rpl32*), two encode DNA-directed RNA polymerase subunits (*rpoB* and *rpoC2)*, and two encode protein translocons on the inner chloroplast membrane (*ycf1* and *ycf2*) (Allen et al., 2011). The M2a x M1a and M8 x M8a contrasts, which are more rigorous in identification and identified four genes (*ndhF*, *rpoC2*, *ycf1*, and *ycf2*) under local positive selection (**Supplementary Table S8**) (Yang and Swanson, 2002; Swanson et al., 2003). The *ndhF* gene encodes NADH dehydrogenase F and is present in most vascular plants. Positive selection of this gene is associated with adaptation to greater light incidence at high altitudes in *Silene vulgaris* (Caryophyllaceae) and salt stress in species of the genus *Limonium* (Plumbaginaceae), suggesting that these loci under positive selection may be related to adaptation in Sapindaceae (Krüger et al., 2019; Darshetkar et al., 2021). The gene rpoC2 is a crucial gene for the transcription of photosynthesis genes, and mutations in these genes can confer the albino phenotype(Park et al., 2023). The *ycf1* and *ycf2* are essential plant genes for photosynthetic protein import and plant survival (Kikuchi et al., 2013; Kikuchi et al., 2018). Previous studies also showed local positive selection in the *ycf1* and *ycf2* genes, both in angiosperm and gimonosperm, related to adaptation to environmental changes (Fan et al., 2018; Zeb et al., 2019). In addition, previous studies without considering the phylogeny indicated positive selection of the ycf2 gene and may, in addition to having loci on positive selection, present clades with a more significant amount of non-synonymous mutations (Saina et al., 2018; Dong et al., 2021).

Previous phylogenies reported for the genus *Serjania* using data from molecular markers (ITS and trnL intron) indicated the formation of a paraphyletic genus with the inclusion of species from the genera *Balsas*, *Chimborazoa* and *Houssayanthus* (Acevedo-Rodríguez et al., 2017). Thus, using molecular information as support, Acevedo-Rodríguez et al. (2017) suggest that the group has changed to include the genera *Balsas*, *Chimborazoa*, and *Houssayanthus* in *Serjania*, forming a monophyletic group. Speciation events within the genus have probably occurred during the Neogene, resulting in over 230 species distributed throughout the Neotropics. The high level of diversification may be related to the ability to disperse seeds by wind (Muellner-Riehl et al., 2016; Acevedo-Rodríguez et al., 2017; Buerki et al., 2021) and the emergence of climbing species on lianas. South American Sapindaceae species, especially lianas, show a distribution across Antarctica that occurred during the Middle Eocene (about 44 million years ago) (Buerki et al., 2021) and diversified about 18.5-19 million years ago (Jud et al., 2021).

Investigation of nucleotide diversity among families and subfamilies allowed the identification of hotspots of genetic diversity, which can be used as molecular markers to resolve intraspecific relationships and as barcoding markers. In general, intergenic spaces show higher nucleotide diversity, while gene regions are more conserved in species, results like those found by the Percentage of variable characters in embryophytes (Zhang et al., 2011; Fan et al., 2018; Zeb et al., 2019). Our results indicate 10 and 11 nucleotide diversity hotspots for Sapindaceae and Sapindoideae; among these six hotspots (*tRNA-Lys* — *rps16*, *ndhC* – *tRNA-Val*, *petA* – *psbJ*, *ndhF*, *rpl32* – *ccsA*, and *ycf1*) are in common for both tribe and family (**Figures 6A, B**). Other studies evaluating nucleotide diversity also indicate the petA-psbJ, rpl32-cssA, ndhF, and ycf1 regions as highly diverse regions, so we strongly suggest using these regions as molecular markers of the group (Zhou et al., 2016; Ma et al., 2019; Dong et al., 2021). Only the *ndhF* and *ycf1* genes showed high nucleotide diversity, genes in which local positive selection was also found (**Figures 6A, B**, and **Supplementary Table S8**), which have already been described and used as molecular markers capable of distinguishing angiosperm and gymnosperm species, with great potential for the family of this study (Dong et al., 2015; Fan et al., 2018; Zeb et al., 2019; Amar, 2020; Ramírez-Barahona et al., 2020; Dong et al., 2021).

The availability of data from chloroplast genomes for the *Serjania* genus can help develop future molecular markers specific to this genus, which can be applied to the definition of interspecific relationships in the genus. However, a more significant number of genomes from phylogenetically close species is required. Although only one chloroplast genome of the genus *Serjania* has been described, the two gene regions have the potential as markers for phylogenetic studies. They can be used to clarify evolutionary issues of the genus. These include modifying vegetational habits associated with the diversification of the group in lianas and fruit shape (Jud et al., 2021). In addition, they have the potential to be used as markers and DNA barcodes in the molecular taxonomic identification of species, providing very useful tools in the certification of natural medicines, as is the case with species of the genus *Serjania*.

## 5 Conclusion

Our study provides the first assembly and annotation of a chloroplast genome of a species from the tribe Paullinieae (Sapindaceae), *Serjania erecta*. Three factors are associated with variation in the number of genes in the chloroplast genomes of sapindaceae: (1) expansion events in the IR, (2) pseudogenization of the *rps2* gene, and (3) absence and duplication of genes of tRNAs. Protein-coding genes are highly conserved in the Sapindaceae family, and only the *psaI* gene is evolving under neutrality. We recommend six regions (*tRNA-Lys — rps16, ndhC – tRNA-Val, petA – psbJ, ndhF, rpl32 – ccsA, and ycf1*) with potential used as a marker for both Sapidaceae and Sapindoideae. The genes *ycf1* and *ndhF*, in addition to showing high nucleotide diversity, also show positive local selection. Our work provides evidence that the *ycf1* and *ndhF* genes may be the most suitable markers for phylogenetic studies of the subfamily Sapindoideae and may contribute to the resolution of taxonomic uncertainties in this group.

## Data availability statement

The datasets presented in this study can be found in online repositories. The names of the repository/repositories and accession number(s) can be found below: https://www.ncbi.nlm.nih.gov/, SRX18977468, https://www.ncbi.nlm.nih.gov/, NC_072944.1.

## Author contributions

LCJC: Data curation, Formal Analysis, Investigation, Methodology, Visualization, Writing – original draft, Writing – review & editing. MS: Formal Analysis, Methodology, Software, Writing – review & editing. LRC: Formal Analysis, Investigation, Software, Writing – review & editing. RD: Formal Analysis, Software, Visualization, Writing – review & editing. RB-F: Formal Analysis, Investigation, Writing – review & editing. CT: Formal Analysis, Writing – review & editing. CS-N: Data curation, Formal Analysis, Investigation, Resources, Writing – review & editing. BB: Investigation, Resources, Writing – review & editing. AS: Resources, Writing – review & editing. JD-f: Conceptualization, Supervision, Writing – review & editing. MT: Conceptualization, Funding acquisition, Project administration, Supervision, Writing – review & editing. RN: Conceptualization, Data curation, Project administration, Supervision, Writing – original draft, Writing – review & editing.

## References

[B1] Acevedo-Rodriguez P. (1990). Distributional patterns in Brazilian serjania (Sapindaceae). Acta Bot. Brasilica 4, 69–82. doi: 10.1590/s0102-33061990000100005

[B2] Acevedo-Rodríguez P. Welzen P. C. Van, Adema F. van der Ham R. W. J. M. (2010). Sapindaceae. Kubitzki, K. (eds) *Flowering Plants. Eudicots. The Families and Genera of Vascular Plants* . 10, 357–407. doi: 10.1007/978-3-642-14397-7_17

[B3] Acevedo-Rodríguez P. Wurdack K. J. Ferrucci M. S. Johnson G. Dias P. Coelho R. G.. (2017). Generic relationships and classification of tribe paullinieae (Sapindaceae) with a new concept of supertribe paulliniodae. Syst. Bot. 42, 96–114. doi: 10.1600/036364417X694926

[B4] Allen J. F. de Paula W. B. M. Puthiyaveetil S. Nield J. (2011). A structural phylogenetic map for chloroplast photosynthesis. Trends Plant Sci. 16, 645–655. doi: 10.1016/j.tplants.2011.10.004 22093371

[B5] Amar M. H. (2020). ycf1-ndhF genes, the most promising plastid genomic barcode, sheds light on phylogeny at low taxonomic levels in Prunus persica. J. Genet. Eng. Biotechnol. 18, 1–10. doi: 10.1186/s43141-020-00057-3 PMC7427673 32797323

[B6] Bausher M. G. Singh N. D. Lee S. B. Jansen R. K. Daniell H. (2006). The complete chloroplast genome sequence of Citrus sinensis (L.) Osbeck var “Ridge Pineapple”: Organization and phylogenetic relationships to other angiosperms. BMC Plant Biol. 6, 1–12. doi: 10.1186/1471-2229-6-21 PMC1599732 17010212

[B7] Beier S. Thiel T. Münch T. Scholz U. Mascher M. (2017). MISA-web: A web server for microsatellite prediction. Bioinformatics 33, 2583–2585. doi: 10.1093/bioinformatics/btx198 28398459 PMC5870701

[B8] Buerki S. Callmander M. W. Acevedo-Rodriguez P. Lowry P. P. Munzinger J. Bailey P.. (2021). An updated infra-familial classification of Sapindaceae based on targeted enrichment data. Am. J. Bot. 108, 1234–1251. doi: 10.1002/ajb2.1693 34219219 PMC8361682

[B9] Buerki S. Forest F. Acevedo-Rodríguez P. Callmander M. W. Nylander J. A. A. Harrington M.. (2009). Plastid and nuclear DNA markers reveal intricate relationships at subfamilial and tribal levels in the soapberry family (Sapindaceae). Mol. Phylogenet. Evol. 51, 238–258. doi: 10.1016/j.ympev.2009.01.012 19405193

[B10] Buerki S. Lowry P. P. Alvarez N. Razafimandimbison S. G. Küpfer P. Callmander M. W. (2010). Phylogeny and circumscription of Sapindaceae revisited: Molecular sequence data, morphology and biogeography support recognition of a new family, Xanthoceraceae. Plant Ecol. Evol. 143, 148–159. doi: 10.5091/plecevo.2010.437

[B11] CBOL Plant Working Group (2009). A DNA barcode for land plants. Proc. Natl. Acad. Sci. U.S.A. 106, 12794–12797. doi: 10.1073/pnas.0905845106 19666622 PMC2722355

[B12] Chase M. W. Christenhusz M. J. M. Fay M. F. Byng J. W. Judd W. S. Soltis D. E.. (2016). An update of the Angiosperm Phylogeny Group classification for the orders and families of flowering plants: APG IV. Botanical J. Linn. Soc. 181, 1–20. doi: 10.1111/boj.12385

[B13] Chen M. Zhang H. Jiang M. (2019). The complete chloroplast genome sequence of Acer cinnamomifolium (Aceraceae), a plant species endemic to China. Mitochondrial DNA B Resour. 4, 3450–3451. doi: 10.1080/23802359.2019.1674211 33366034 PMC7707229

[B501] Christopher D. A. Cushman J. C. Price C. A. Hallick R. B. (1988). Organization of ribosomal protein genes rp123, rpl2, rpsl9, rpl22 and rps3 on the Euglena gracilis chloroplast genome. Curr. Genet. 14, 275–285. doi: 10.1007/BF00376748 3143485

[B14] Darling A. C. E. Mau B. Blattner F. R. Perna N. T. (2004). Mauve: Multiple alignment of conserved genomic sequence with rearrangements. Genome Res. 14, 1394–1403. doi: 10.1101/gr.2289704 15231754 PMC442156

[B15] Darshetkar A. M. Maurya S. Lee C. Bazarragchaa B. Batdelger G. Janchiv A.. (2021). Plastome analysis unveils Inverted Repeat (IR) expansion and positive selection in Sea Lavenders (Limonium, Plumbaginaceae, Limonioideae, Limonieae). PhytoKeys 175, 89–107. doi: 10.3897/phytokeys.175.61054 33867801 PMC8032645

[B16] Dierckxsens N. Mardulyn P. Smits G. (2017). NOVOPlasty: *De novo* assembly of organelle genomes from whole genome data. Nucleic Acids Res. 45, 1–9. doi: 10.1093/nar/gkw955 PMC5389512 28204566

[B17] Dong F. Lin Z. Lin J. Ming R. Zhang W. (2021). Chloroplast genome of rambutan and comparative analyses in sapindaceae. Plants 10, 1–15. doi: 10.3390/plants10020283 PMC7912957 33540810

[B18] Dong W. Xu C. Li C. Sun J. Zuo Y. Shi S.. (2015). ycf1, the most promising plastid DNA barcode of land plants. Sci. Rep. 5 8348, 1–5. doi: 10.1038/srep08348 25672218 PMC4325322

[B19] Doyle J. J. Doyle J. L. (1987). A rapid DNA isolation procedure for small quantities of fresh leaf tissue. Phytochem. Bull. 19, 11–15.

[B20] Dugas D. V. Hernandez D. Koenen E. J. M. Schwarz E. Straub S. Hughes C. E.. (2015). Mimosoid legume plastome evolution: IR expansion, tandem repeat expansions, and accelerated rate of evolution in clpP. Sci. Rep. 5, 1–13. doi: 10.1038/srep16958 PMC4655330 26592928

[B21] Fan W. B. Wu Y. Yang J. Shahzad K. Li Z. H. (2018). Comparative chloroplast genomics of dipsacales species: Insights into sequence variation, adaptive evolution, and phylogenetic relationships. Front. Plant Sci. 9. doi: 10.3389/fpls.2018.00689 PMC5974163 29875791

[B22] Ferrucci M. S. Acevedo-Rodríguez P. (2005). Three new species of Serjania (Sapindaceae) from south America. Syst. Bot. 30, 153–162. doi: 10.1600/0363644053661904

[B23] Gantt J. S. Baldauf S. L. Calie P. J. Weeden N. F. Palmer J. D. (1991). Transfer of rpl22 to the nucleus greatly preceded its loss from the chloroplast and involved the gain of an intron. EMBO J. 10, 3073–3078. doi: 10.1002/j.1460-2075.1991.tb07859.x 1915281 PMC453023

[B24] Gomig F. Pietrovski E. F. Guedes A. Dalmarco E. M. Calderari M. T. Guimarães C. L.. (2008). Topical anti-inflammatory activity of Serjania erecta Radlk (Sapindaceae) extracts. J. Ethnopharmacol. 118, 220–224. doi: 10.1016/j.jep.2008.03.017 18513901

[B25] Hildebrand M. Hallick R. B. Passavant C. W. Bourque D. P. (1988). Trans-splicing in chloroplasts: The rpsl2 loci of Nicotiana tabacum. Proc. Natl. Acad. Sci. U.S.A. 85, 372–376. doi: 10.1073/pnas.85.2.372 PMC279550 3422433

[B26] Hiruma-Lima C. A. Castelo A. P. C. Arruda B. N. Coelho R. G. Honda N. K. Ferrazoli C.. (2009). Gastroprotective effect of Serjania erecta Radlk (Sapindaceae): Involvement of sensory neurons, endogenous nonprotein sulfhydryls, and nitric oxide. J. Med. Food 12, 1411–1415. doi: 10.1089/jmf.2008.0269 20041803

[B27] Jansen R. K. Ruhlman T. A. (2012). Plastid genomes of seed plants. Genomics of Chloroplasts and Mitochondria, Advances in Photosynthesis and Respiration. 35, 103–126. doi: 10.1007/978-94-007-2920-9_5

[B28] Jud N. A. Allen S. E. Nelson C. W. Bastos C. L. Chery J. G. (2021). Climbing since the early Miocene: The fossil record of Paullinieae (Sapindaceae). PloS One 16, 1–22. doi: 10.1371/journal.pone.0248369 PMC8026063 33826635

[B29] Kalyaanamoorthy S. Minh B. Q. Wong T. K. F. Von Haeseler A. Jermiin L. S. (2017). ModelFinder: Fast model selection for accurate phylogenetic estimates. Nat. Methods 14, 587–589. doi: 10.1038/nmeth.4285 28481363 PMC5453245

[B30] Kang B. C. Bae S. J. Lee S. Lee J. S. Kim A. Lee H.. (2021). Chloroplast and mitochondrial DNA editing in plants. Nat. Plants 7, 899–905. doi: 10.1038/s41477-021-00943-9 34211132 PMC8289734

[B31] Katoh K. Standley D. M. (2013). MAFFT multiple sequence alignment software version 7: Improvements in performance and usability. Mol. Biol. Evol. 30, 772–780. doi: 10.1093/molbev/mst010 23329690 PMC3603318

[B32] Kearse M. Moir R. Wilson A. Stones-Havas S. Cheung M. Sturrock S.. (2012). Geneious Basic: An integrated and extendable desktop software platform for the organization and analysis of sequence data. Bioinformatics 28, 1647–1649. doi: 10.1093/bioinformatics/bts199 22543367 PMC3371832

[B33] Kent W. J. (2002). BLAT —The BLAST -like alignment tool. Genome Res. 12, 656–664. doi: 10.1101/gr.229202 11932250 PMC187518

[B34] Kikuchi S. Asakura Y. Imai M. Nakahira Y. Kotani Y. Hashiguchi Y.. (2018). A Ycf2-FtsHi heteromeric AAA-ATPase complex is required for chloroplast protein import. Plant Cell 30, 2677–2703. doi: 10.1105/tpc.18.00357 30309901 PMC6305978

[B35] Kikuchi S. Bédard J. Hirano M. Hirabayashi Y. Oishi M. Imai M.. (2013). Uncovering the protein translocon at the chloroplast inner envelope membrane. Sci. (1979) 339, 571–574. 10.1126/science.1229262 23372012

[B36] Köhler M. Reginato M. Souza-Chies T. T. Majure L. C. (2020). Insights into chloroplast genome evolution across opuntioideae (Cactaceae) reveals robust yet sometimes conflicting phylogenetic topologies. Front. Plant Sci. 11. doi: 10.3389/fpls.2020.00729 PMC7317007 32636853

[B37] Krüger M. Abeyawardana O. A. J. Juříček M. Krüger C. Štorchová H. (2019). Variation in plastid genomes in the gynodioecious species Silene vulgaris. BMC Plant Biol. 19, 1–15. doi: 10.1186/s12870-019-2193-0 PMC6921581 31856730

[B38] Kurtz S. Choudhuri J. V. Ohlebusch E. Schleiermacher C. Stoye J. Giegerich R. (2001). REPuter: the manifold applications of repeat analysis on a genomic scale. Nucleic Acids Res. 29, 4633–4642. doi: 10.1093/nar/29.22.4633 PMC92531 11713313

[B39] Laslett D. Canback B. (2004). ARAGORN, a program to detect tRNA genes and tmRNA genes in nucleotide sequences. Nucleic Acids Res. 32, 11–16. doi: 10.1093/nar/gkh152 14704338 PMC373265

[B40] Leebens-Mack J. Raubeson L. A. Cui L. Kuehl J. V. Fourcade M. H. Chumley T. W.. (2005). Identifying the basal angiosperm node in chloroplast genome phylogenies: Sampling one’s way out of the Felsenstein zone. Mol. Biol. Evol. 22, 1948–1963. doi: 10.1093/molbev/msi191 15944438

[B43] Li Z. Long H. Zhang L. Liu Z. Cao H. Shi M.. (2017). The complete chloroplast genome sequence of tung tree (Vernicia fordii): Organization and phylogenetic relationships with other angiosperms. Sci. Rep. 7, 1–11. doi: 10.1038/s41598-017-02076-6 PMC5431841 28500291

[B42] Li P. Lou G. Cai X. Zhang B. Cheng Y. Wang H. (2020b). Comparison of the complete plastomes and the phylogenetic analysis of Paulownia species. Sci. Rep. 10, 1–9. doi: 10.1038/s41598-020-59204-y PMC7010769 32042041

[B41] Li C. Zhao Y. Xu Z. Yang G. Peng J. Peng X. (2020a). Initial characterization of the chloroplast genome of vicia sepium, an important wild resource plant, and related inferences about its evolution. Front. Genet. 11. doi: 10.3389/fgene.2020.00073 PMC7044246 32153639

[B44] Lohse M. Drechsel O. Kahlau S. Bock R. (2013). OrganellarGenomeDRAW–a suite of tools for generating physical maps of plastid and mitochondrial genomes and visualizing expression data sets. Nucleic Acids Res. 41, W575–W581. doi: 10.1093/nar/gkt289 PMC3692101 23609545

[B45] Ma Q. Wang Y. Zhu L. Bi C. Li S. Li S.. (2019). Characterization of the complete chloroplast genome of acer truncatum bunge (Sapindales: Aceraceae): A new woody oil tree species producing nervonic acid. BioMed. Res. Int. 2019, 1–13. doi: 10.1155/2019/7417239 PMC6925723 31886246

[B46] Margulis L. Bermudes D. (1985). Symbiosis as a mechanism of evolution: status of cell symbiosis theory. Symbiosis 1, 101–124. 11543608

[B47] Millen R. S. Olmstead R. G. Adams K. L. Palmer J. D. Lao N. T. Heggie L.. (2001) Many Parallel Losses of infA from Chloroplast DNA during Angiosperm Evolution with Multiple Independent Transfers to the Nucleus. Available at: www.plantcell.org. 10.1105/tpc.13.3.645 PMC135507 11251102

[B48] Moore M. J. Soltis P. S. Bell C. D. Burleigh J. G. Soltis D. E. (2010). Phylogenetic analysis of 83 plastid genes further resolves the early diversification of eudicots. Proc. Natl. Acad. Sci. U.S.A. 107, 4623–4628. doi: 10.1073/pnas.0907801107 20176954 PMC2842043

[B49] Muellner-Riehl A. N. Weeks A. Clayton J. W. Buerki S. Nauheimer L. Chiang Y. C.. (2016). Molecular phylogenetics and molecular clock dating of Sapindales based on plastid rbcL, atpB and trnL-trnF DNA sequences. Taxon 65, 1019–1036. doi: 10.12705/655.5

[B50] Nei M. Kumar S. (2000). Molecular evolution and phylogenetics (New York, New York, USA: Oxford University Press).

[B51] Nguyen L. T. Schmidt H. A. Von Haeseler A. Minh B. Q. (2015). IQ-TREE: A fast and effective stochastic algorithm for estimating maximum-likelihood phylogenies. Mol. Biol. Evol. 32, 268–274. doi: 10.1093/molbev/msu300 25371430 PMC4271533

[B52] Novoa E. M. Pavon-Eternod M. Pan T. Ribas De Pouplana L. (2012). A role for tRNA modifications in genome structure and codon usage. Cell 149, 202–213. doi: 10.1016/j.cell.2012.01.050 22464330

[B53] Okonechnikov K. Golosova O. Fursov M. Varlamov A. Vaskin Y. Efremov I.. (2012). Unipro UGENE: A unified bioinformatics toolkit. Bioinformatics 28, 1166–1167. doi: 10.1093/bioinformatics/bts091 22368248

[B54] Park H. S. Jeon J. H. Cho W. Lee Y. Park J. Y. Kim J.. (2023). High-throughput discovery of plastid genes causing albino phenotypes in ornamental chimeric plants. Hortic. Res. 10, 1–11. doi: 10.1093/hr/uhac246 PMC9832966 36643742

[B55] Plöchinger M. Torabi S. Rantala M. Tikkanen M. Suorsa M. Jensen P. E.. (2016). The low molecular weight protein psai stabilizes the light-harvesting complex II docking site of photosystem I. Plant Physiol. 172, 450–463. doi: 10.1104/pp.16.00647 27406169 PMC5074619

[B57] Ramírez-Barahona S. Sauquet H. Magallón S. (2020). The delayed and geographically heterogeneous diversification of flowering plant families. Nat. Ecol. Evol. 4, 1232–1238. doi: 10.1038/s41559-020-1241-3 32632260

[B56] R Core Team (2020). R: A language and environment for statistical computing (Vienna, Austria: R Foundation for Statistical Computing).

[B58] Rodríguez-Ezpeleta N. Brinkmann H. Burey S. C. Roure B. Burger G. Löffelhardt W.. (2005). Monophyly of primary photosynthetic eukaryotes: Green plants, red algae, and glaucophytes. Curr. Biol. 15, 1325–1330. doi: 10.1016/j.cub.2005.06.040 16051178

[B59] Rozas J. Ferrer-Mata A. Sanchez-DelBarrio J. C. Guirao-Rico S. Librado P. Ramos-Onsins S. E.. (2017). DnaSP 6: DNA sequence polymorphism analysis of large data sets. Mol. Biol. Evol. 34, 3299–3302. doi: 10.1093/molbev/msx248 29029172

[B60] Saina J. K. Gichira A. W. Li Z. Z. Hu G. W. Wang Q. F. Liao K. (2018). The complete chloroplast genome sequence of Dodonaea viscosa: comparative and phylogenetic analyses. Genetica 146, 101–113. doi: 10.1007/s10709-017-0003-x 29170851

[B61] Sato N. (2020). Complex origins of chloroplast membranes with photosynthetic machineries: multiple transfers of genes from divergent organisms at different times or a single endosymbiotic event? J. Plant Res. 133, 15–33. doi: 10.1007/s10265-019-01157-z 31811433 PMC6946739

[B62] Schöttler M. A. Thiele W. Belkius K. Bergner S. V. Flügel C. Wittenberg G.. (2017). The plastid-encoded PsaI subunit stabilizes photosystem i during leaf senescence in tobacco. J. Exp. Bot. 68, 1137–1155. doi: 10.1093/jxb/erx009 28180288 PMC5429015

[B63] Sobreiro M. B. Vieira L. D. Nunes R. Novaes E. Coissac E. Silva-Junior O. B.. (2020). Chloroplast genome assembly of Handroanthus impetiginosus: comparative analysis and molecular evolution in Bignoniaceae. Planta 252, 1–16. doi: 10.1007/s00425-020-03498-9 33098500

[B64] Somner G. V. Ferrucci M. S. Acevedo-Rodríguez P. (2015). Serjania in Lista de Espécies da Flora do Brasil. Jardim Botânico do Rio Janeiro. Available at: https://floradobrasil.jbrj.gov.br/F (Accessed March 19, 2023).

[B65] Souza U. J. B. d. Nunes R. Targueta C. P. Diniz-Filho J. A. F. Telles M. P. d. C. (2019). The complete chloroplast genome of Stryphnodendron adstringens (Leguminosae - Caesalpinioideae): comparative analysis with related Mimosoid species. Sci. Rep. 9, 1–12. doi: 10.1038/s41598-019-50620-3 PMC6775074 31578450

[B500] Steinmann V. W. Ferrucci M. S. Maya-Lastra C. A. (2022). Phylogenetics of Serjania (Sapindaceae-Paullinieae), with emphasis on fruit evolution and the description of a new species from Michoacán, Mexico. Syst. Biodivers. 20, 1–21. doi: 10.1080/14772000.2022.2030425 36970113

[B66] Swanson W. J. Nielsen R. Yang Q. (2003). Pervasive adaptive evolution in mammalian fertilization proteins. Mol. Biol. Evol. 20, 18–20. doi: 10.1093/oxfordjournals.molbev.a004233 12519901

[B67] Talavera G. Castresana J. (2007). Improvement of phylogenies after removing divergent and ambiguously aligned blocks from protein sequence alignments. Syst. Biol. 56, 564–577. doi: 10.1080/10635150701472164 17654362

[B68] Tillich M. Lehwark P. Pellizzer T. Ulbricht-Jones E. S. Fischer A. Bock R.. (2017). GeSeq - Versatile and accurate annotation of organelle genomes. Nucleic Acids Res. 45, W6–W11. doi: 10.1093/nar/gkx391 28486635 PMC5570176

[B69] Urdampilleta J. D. Ferrucci M. S. Vanzela A. L. L. (2012). Cytogenetic studies in South American species of Serjania (Sapindaceae: Paullinieae). Plant Biosyst. 146, 835–846. doi: 10.1080/11263504.2012.705349

[B70] Vaidya G. Lohman D. J. Meier R. (2011). SequenceMatrix: Concatenation software for the fast assembly of multi-gene datasets with character set and codon information. Cladistics 27, 171–180. doi: 10.1111/j.1096-0031.2010.00329.x 34875773

[B71] Varshney R. K. Graner A. Sorrells M. E. (2005). Genic microsatellite markers in plants: Features and applications. Trends Biotechnol. 23, 48–55. doi: 10.1016/j.tibtech.2004.11.005 15629858

[B72] Wang W. Chen S. Zhang X. (2018). Whole-Genome comparison reveals divergent IR borders and mutation hotspots in chloroplast genomes of herbaceous bamboos (Bambusoideae: Olyreae). Molecules 23, 1–20. doi: 10.3390/molecules23071537 PMC6099781 29949900

[B73] Wang Y. H. Qu X. J. Chen S. Y. Li D. Z. Yi T. S. (2017). Plastomes of Mimosoideae: structural and size variation, sequence divergence, and phylogenetic implication. Tree Genet. Genomes 13, 1–18. doi: 10.1007/s11295-017-1124-1

[B74] Wheeler T. J. Eddy S. R. (2013). Nhmmer: DNA homology search with profile HMMs. Bioinformatics 29, 2487–2489. doi: 10.1093/bioinformatics/btt403 23842809 PMC3777106

[B75] Wicke S. Schneeweiss G. M. dePamphilis C. W. Müller K. F. Quandt D. (2011). The evolution of the plastid chromosome in land plants: Gene content, gene order, gene function. Plant Mol. Biol. 76, 273–297. doi: 10.1007/s11103-011-9762-4 21424877 PMC3104136

[B76] Wu Y. Liu F. Yang D. G. Li W. Zhou X. J. Pei X. Y.. (2018). Comparative chloroplast genomics of Gossypium species: Insights into repeat sequence variations and phylogeny. Front. Plant Sci. 9. doi: 10.3389/fpls.2018.00376 PMC5871733 29619041

[B77] Xiao-Ming Z. Junrui W. Li F. Sha L. Hongbo P. Lan Q.. (2017). Inferring the evolutionary mechanism of the chloroplast genome size by comparing whole-chloroplast genome sequences in seed plants. Sci. Rep. 7, 1–10. doi: 10.1038/s41598-017-01518-5 PMC5431534 28484234

[B78] Yang Z. (2007). PAML 4: Phylogenetic analysis by maximum likelihood. Mol. Biol. Evol. 24, 1586–1591. doi: 10.1093/molbev/msm088 17483113

[B79] Yang Z. Swanson W. J. (2002). Codon-substitution models to detect adaptive evolution that account for heterogeneous selective pressures among site classes. Mol. Biol. Evol. 19, 49–57. doi: 10.1093/oxfordjournals.molbev.a003981 11752189

[B80] Zeb U. Dong W.-L. Zhang T.-T. Wang R.-N. Shahzad K. Ma X.-F.. (2019). Comparative plastid genomics of Pinus species: Insights into sequence variations and phylogenetic relationships. JSE J. Syst. Evol. 00, 1–15. doi: 10.1002/jse.12492

[B81] Zhang Y. J. Ma P. F. Li D. Z. (2011). High-throughput sequencing of six bamboo chloroplast genomes: Phylogenetic implications for temperate woody bamboos (Poaceae: Bambusoideae). PloS One 6, 1–16. doi: 10.1371/journal.pone.0020596 PMC3105084 21655229

[B82] Zhou T. Chen C. Wei Y. Chang Y. Bai G. Li Z.. (2016). Comparative transcriptome and chloroplast genome analyses of two related dipteronia species. Front. Plant Sci. 7. doi: 10.3389/fpls.2016.01512 PMC5061820 27790228

