# Supplementary material for: Chloroplast genome assembly of Serjania erecta Raldk: comparative analysis reveals gene number variation and selection in protein-coding plastid genes of Sapindaceae
Source: Front Plant Sci. 2023 Sep 26;14:1258794. doi: 10.3389/fpls.2023.1258794 (PMC10562606; doi:10.3389/fpls.2023.1258794)
Supplement: Supplementary file 1 [file DataSheet_1.pdf]

Supplementary Material.

1 Supplementary Figures and Tables

1.1 Supplementary Figures

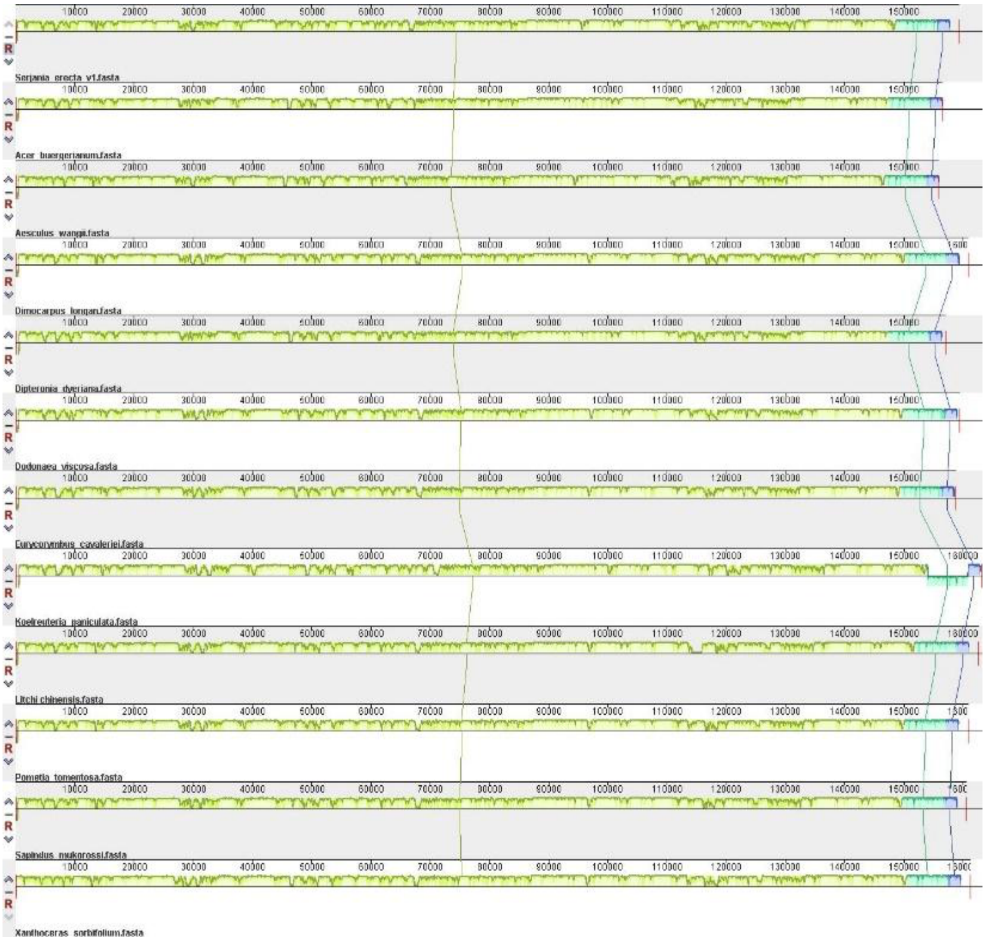

Supplementary Figure S1. Progressive alignment of the chloroplast genomes of the twelve species of the Sapindaceae family.

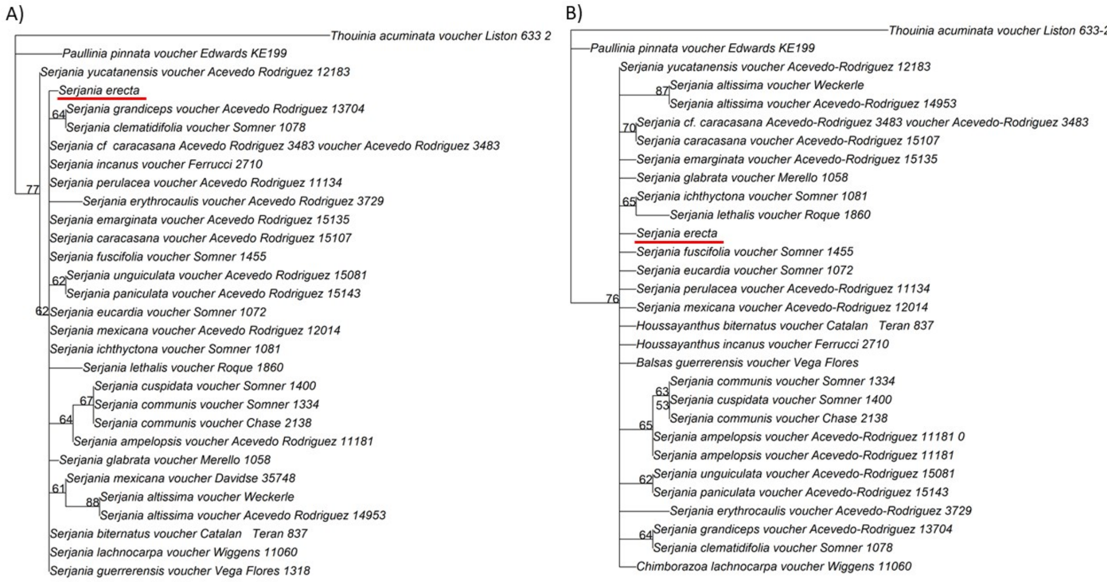

Supplementary Figure S2. The maximum likelihood (ML) phylogenetic tree for the genus *Serjania*, based on molecular markers. Data obtained from studies by Buerki et al. (2009), Buerki et al. (2010) and Acevedo-Rodríguez et al. (2017). A) ML phylogenetic tree using molecular markers *matK*, *rpoB*, *trnD-trnT*, *trnK-matK*, *trnL*(intron), and *trnL-trnF*. B) ML phylogenetic tree using the tag *trnL*(intron).

1.2 Supplementary Table

Supplementary Table S1. The Genbank accession number of the chloroplast genomes used in assembling the phylogenetic tree using protein-coding sequences.

Species	family	ID
Serjania erecta	Sapindaceae	In this work
Acer buergerianum	Sapindaceae	NC_034744.1
Acer truncatum	Sapindaceae	NC_037211.1
Acer longipes	Sapindaceae	NC_049126.1
Aesculus chinensis	Sapindaceae	NC_046788.1
Aesculus wangii	Sapindaceae	NC_035955.1
Dimocarpus longan	Sapindaceae	NC_037447.1
Dipteronia sinensis	Sapindaceae	NC_029338.1
Dipteronia dyeriana	Sapindaceae	NC_031899.1
Dodonaea viscosa	Sapindaceae	NC_036099.1
Eurycorymbus cavaleriei	Sapindaceae	NC_037443.1
Koelreuteria paniculata	Sapindaceae	NC_037176.1
Litchi chinensis	Sapindaceae	NC_035238.1
Pometia tomentosa	Sapindaceae	NC_048999.1
Sapindus mukorossi	Sapindaceae	NC_025554.1
Xanthoceras sorbifolium	Sapindaceae	NC_037448.1
Khaya senegalensis	Meliacea	NC_037362.1
Cedrela odorata	Meliacea	NC_037251.1
Citrus sinensis	Rutaceae	NC_008334.1
Ruta graveolens	Rutaceae	NC_045946.1
Mangifera indica	Anacardiacea e	NC_035239.1
Boswellia sacra	Burseraceae	NC_029420.1
Gossypium hirsutum	Malvaceae	NC_007944.1

Supplementary Table S2. Genbank access numbers of sequences used for phylogenetic analysis of the genus *Serjania.*

Species	Reference	matK	rpoB	trnD-trnT	trnK-matK	trnL intron	trnL-trnF
Serjania altissima	Buerki et al, 2009		EU720840			EU721269	EU721457
Serjania communis	Buerki et al, 2009	EU720640	EU720805	EU720969	EU721077	EU721241	EU721429
Serjania glabrata	Buerki et al, 2009	EU720703	EU720905	EU721010	EU721141	EU721327	EU721515
Serjania mexicana	Buerki et al, 2010	EU720704	EU720906		EU721142		
Serjania altissim	Acevedo-Rodríguez et al, 2017					KX585036	
Serjania ampelopsis	Acevedo-Rodríguez et al, 2017					KX585037	
Serjania caracasana	Acevedo-Rodríguez et al, 2017					KX585038	
Serjania cf. caracasana	Acevedo-Rodríguez et al, 2017					KX585039	
Serjania clematidifolia	Acevedo-Rodríguez et al, 2017					KX585040	
Serjania communis	Acevedo-Rodríguez et al, 2017					KX585041	
Serjania cuspidata	Acevedo-Rodríguez et al, 2017					KX585042	
Serjania emarginata	Acevedo-Rodríguez et al, 2017					KX585043	
Serjania erythrocaulis	Acevedo-Rodríguez et al, 2017					KX585044	
Serjania eucardia	Acevedo-Rodríguez et al, 2017					KX585045	
Serjania fuscifolia	Acevedo-Rodríguez et al, 2017					KX585046	

Serjania grandiceps	Acevedo-Rodríguez et al, 2017					KX585047	
Serjania ichthyoctona	Acevedo-Rodríguez et al, 2017					KX585048	
Serjania lethalis	Acevedo-Rodríguez et al, 2017					KX585049	
Serjania marginata	Acevedo-Rodríguez et al, 2017					KX585051	
Serjania mexicana	Acevedo-Rodríguez et al, 2017					KX585052	
Serjania paniculata	Acevedo-Rodríguez et al, 2017					KX585054	
Serjania perulacea	Acevedo-Rodríguez et al, 2017					KX585055	
Serjania unguiculata	Acevedo-Rodríguez et al, 2017					KX585056	
Serjania yucatanensis	Acevedo-Rodríguez et al, 2017					KX585057	
Chimborazoa lachnocarpa	Acevedo-Rodríguez et al, 2017					KX585014	
Balsas guerrerensis	Acevedo-Rodríguez et al, 2017					KX585005	
Houssayanthus biternatus	Acevedo-Rodríguez et al, 2017					KX585018	
Houssayanthus incanus	Acevedo-Rodríguez et al, 2017					KX585019	
Paullinia pinnata	Buerki et al, 2009	EU720575	EU720730	EU720926	EU721023	EU721167	EU721355
Thouinia acuminata	Buerki et al, 2009	EU720647	EU720814		EU721084	EU721249	EU721437

Supplementary Table S3. Categorization of annotated genes in the chloroplast genome of *Serjania erecta.*

Category	Gene groups	Gene names
Self-replication	Large subunit of ribosomal proteins	rpl2[1]*, rpl14, rpl16[1], rpl20, rpl22*, rpl23*, rpl32, rpl33, rpl36
	Small subunit of ribosomal proteins	rps2, rps3*, rps4, rps7*, rps8, rps11, rps12[1]*, rps14, rps15, rps16[1], rps18, rps19*
	DNA-dependent RNA polymerase	rpoA, rpoB, rpoC1[1], rpoC2
	Ribosomal RNA genes	rrn4.5*, rrn5*, rrn16*, rrn23*
	Transfer RNA genes	trnA-UGC[1]*, trnC-GCA, trnD-GUC, trnE-UUC, trnE-UUC*, trnF-GAA, trnG-GCC, trnH-GUG, trnK-UUU[1], trnL-CAA*, trnL-UAA[1], trnL-UAG, trnM-CAU**, trnN-GUU*, trnP-UGG, trnR-UCU, trnQ-UUG, trnR-ACG*, trnS-GCU, trnS-GGA, trnS-UGA, trnT-CGU[1], trnT-GGU, trnT-UGU, trnV-GAC*, trnV-UAC[1], trnW-CCA, trnY-GUA
Photosynthesis	Photosystem I	psaA, psaB, psaC, psaI, psaJ
	Photosystem II	psbA, psbB, psbC, psbD, psbE, psbF, psbH, psbI, psbJ, psbK, psbL, psbM, psbN, psbT, psbZ
NADH dehydrogenase	NADH dehydrogenase	ndhA[1], ndhB[1]*, ndhC, ndhD, ndhE, ndhF, ndhG, ndhH, ndhI, ndhJ, ndhK
	Cytochrome b/f complex	petA, petB[1], petD[1], petG, petL, petN
	ATP synthase	atpA, atpB, atpE, atpF[1], atpH, atpI
	RubisCo large subunit	rbcL
Other genes	Maturase K	matK
	Envelope membrane protein	cemA
	Subunit of acetyl-CoAcarboxylase	accD
	C-type cytochrome synthesis gene	ccsA
	Protease	clpP[2]
	Conserved hypothetical chloroplast open reading frames	ycf1, ycf2*, ycf3[2], ycf4
Pseudogenes		infA, ycf1

Note: *: duplicated genes; **: genes with 4 copies [1]: genes with one intron; [2]: genes with two introns.

Supplementary Table S4. Codon usage from the chloroplast genome of *Serjania erecta*.

Amino Acid	Codon	Number	Fraction	Amino Acid	Codon	Number	Fraction
Ala	GCA	391	0.27	Leu	CTT	590	0.21
	GCC	229	0.16		TTA	855	0.30
	GCG	195	0.14		TTG	588	0.21
	GCT	621	0.43	Lys	AAA	1136	0.75
Arg	AGA	459	0.28		AAG	384	0.25
	AGG	197	0.12	Met	ATG	628	1.00
	CGA	373	0.23	Phe	TTC	544	0.34
	CGC	141	0.09		TTT	1052	0.66
	CGG	156	0.10	Pro	CCA	293	0.27
	CGT	309	0.19		CCC	250	0.23
Asn	AAC	307	0.23		CCG	173	0.16
	AAT	1032	0.77		CCT	384	0.35
Asp	GAC	228	0.22	Ser	AGC	140	0.07
	GAT	818	0.78		AGT	414	0.20
Cys	TGC	91	0.29		TCA	428	0.21
	TGT	227	0.71		TCC	330	0.16
Gln	CAA	742	0.77		TCG	215	0.10
	CAG	220	0.23		TCT	541	0.26
Glu	GAA	1059	0.74	Thr	ACA	404	0.30
	GAG	369	0.26		ACC	261	0.19
Gly	GGA	718	0.39		ACG	169	0.13
	GGC	188	0.10		ACT	511	0.38
	GGG	358	0.19	Trp	TGG	454	1.00
	GGT	575	0.31	Tyr	TAC	197	0.20
His	CAC	169	0.27		TAT	778	0.80
	CAT	467	0.73	Val	GTA	537	0.37
Ile	ATA	720	0.32		GTC	181	0.12
	ATC	429	0.19		GTG	198	0.14
	ATT	1131	0.50		GTT	543	0.37
Leu	CTA	388	0.14	End	TAA	53	0.58
	CTC	217	0.08		TAG	22	0.24
	CTG	197	0.07		TGA	17	0.18

Supplementary Table S5. Intron size and their respective genes in chloroplast genomes of the Sapindaceae family.

Region	LSC														IR					SCC
Species/Gene	*trnK-UUU*	*rps16*	*trnT-CGU*	*atpF*	*rpoC1*	*ycf3 1*	*ycf3 2*	*trnL-UAA*	*trnV-UAC*	*clpP 1*	*clpP 2*	*petB*	*petD*	*rpl16*	*rpl2*	*ndhB*	*rps12*	*trnE-UUC*	*trnA-UGC*	*ndhA*
Serjania erecta	2556	854	720	787	709	764	744	543	597	671	826	804	743	1056	663	607	541	956	802	1126
Acer buergerianum	2667	812	715	767	692	730	732	515	590	596	853	786	747	1053	664	682	545	957	807	1099
Aesculus wangii	2542	824	720	755	705	744	729	537	588	615	846	804	739	1051	664	681	537	958	840	1101
Dimocarpus longan	2511	839	725	754	717	745	730	536	601	655	874	798	766	1063	663	672	541	956	839	1082
Dipteronia dyeriana	2501	794	715	750	700	733	730	522	593	612	840	797	736	1073	664	682	546	957	807	1085
Dodonaea viscosa	2513	840	absent gene	758	719	824	732	553	591	662	875	816	733	1045	664	680	533	951	803	1106
Eurycorymbus cavaleriei	2498	834	absent gene	762	740	767	732	544	592	658	850	809	728	926	664	680	533	957	810	1089
Koelreuteria paniculata	2527	841	730	764	717	756	741	550	605	641	985	785	757	1074	663	672	541	963	841	522
Litchi chinensis	2511	837	725	756	714	745	735	536	600	655	867	798	812	1042	663	672	541	956	846	1110
Pometia tomentosa	2512	839	725	756	712	746	730	536	605	656	867	796	742	1028	663	672	541	957	840	1078
Sapindus mukorossi	2536	832	730	778	714	766	731	538	606	664	870	793	745	985	663	672	541	952	840	1102
Xanthoceras sorbifolium	2465	823	721	767	700	756	730	543	593	637	907	792	736	1055	664	681	536	961	840	1118

Supplementary Table S6. Nucleotide diversity hotspot regions, mean and median values of nucleotide diversity for 12 chloroplast genomes of the family Sapindaceae.

Regions	Nucleotide diversity (π)
tRNA-Lys-rps16	0.0981
atpI–rps2	0.0844
rpoB-tRNA-Asp	0.0817
tRNA-Tyr-psbD	0.0837
psbZ	0.0865
ndhC-tRNA-Val	0.1052
petA-psbJ	0.0963
ndhF	0.1089
rpl32-ccsA	0.1133
ycf1	0.1473
Median	0.0357
Average	0.0380
Standard deviation	0.0274

Supplementary Table S7. Nucleotide diversity hotspot regions, mean and median values of nucleotide diversity for 6 chloroplast genomes of the subfamily Sapindoideae.

Regions	Nucleotide diversity (π)
tRNA-Lys-rps16	0.0717
rps16-tRNA-Gln	0.0680
tRNA-Ser-tRNA-Gly	0.0597
tRNA-Cys-psbM	0.0638
ndhC-tRNA-Val	0.0816
ycf4-cemA	0.0683
petA-psbJ	0.0704
psbE-petL	0.0681
ndhF	0.0696
rpl32-ccsA	0.0807
ycf1	0.0974
Median	0.0227
Average	0.0244
Standard deviation	0.0187

Supplementary Table S8. Local selection in Sapindaceae chloroplast genome protein coding genes.

Gene	ka/ks	LH1	NP1	LH2	NP2	LH7	NP7	LH8	NP8	LH8a	NP8a	M2xM1	P_M2xM1	FDR_M2xM1	M8xM7	P_M8xM7	FDR_M8xM7	M8axM8	P_M8axM8	FDR_M8axM8
accD	0.35	-4073.91	25	-4073.10	27	-4074.18	25	-4072.76	27	-4073.88	26	1.62	0.44	1.00	2.84	0.24	0.83	2.25	0.33	1.00
**atpA**	0.09	-3274.52	25	-3273.30	27	-3278.74	25	-3273.71	27	-3274.46	26	2.44	0.30	1.00	10.06	0.01	**0.05**	1.50	0.47	1.00
atpB	0.08	-3201.72	25	-3201.72	27	-3202.68	25	-3201.67	27	-3201.67	26	0.00	1.00	1.00	2.03	0.36	1.00	0.00	1.00	1.00
atpE	0.11	-823.44	25	-823.44	27	-823.83	25	-823.44	27	-823.46	26	0.00	1.00	1.00	0.78	0.68	1.00	0.04	0.98	1.00
atpF	0.33	-1297.96	25	-1297.96	27	-1297.97	25	-1297.96	27	-1297.96	26	0.00	1.00	1.00	0.03	0.99	1.00	0.00	1.00	1.00
atpH	0.00	-461.48	25	-461.48	27	-461.48	25	-461.48	27	-461.48	26	0.00	1.00	1.00	0.00	1.00	1.00	0.00	1.00	1.00
atpI	0.11	-1638.28	25	-1638.28	27	-1638.28	25	-1638.28	27	-1638.28	26	0.00	1.00	1.00	0.00	1.00	1.00	0.00	1.00	1.00
ccsA	0.51	-3275.11	25	-3275.11	27	-3275.13	25	-3271.92	27	-3276.28	26	0.00	1.00	1.00	6.43	0.04	0.19	8.71	0.01	0.14
cemA	0.30	-1739.06	25	-1739.06	27	-1738.76	25	-1738.76	27	-1738.76	26	0.00	1.00	1.00	0.00	1.00	1.00	0.00	1.00	1.00
**clpP**	0.70	-1659.93	25	-1654.62	27	-1659.94	25	-1654.65	27	-1659.93	26	10.63	0.00	0.06	10.58	0.01	**0.04**	10.56	0.01	0.07

**matK**	0.45	-4928.26	25	-4925.52	27	-4929.94	25	-4925.13	27	-4928.28	26	5.49	0.06	0.47	9.61	0.01	**0.05**	6.29	0.04	0.30
**ndhA**	0.22	-2611.54	25	-2605.86	27	-2613.28	25	-2606.35	27	-2611.56	26	11.35	0.00	0.05	13.87	0.00	**0.01**	10.42	0.01	0.07
ndhB	0.33	-2389.78	25	-2389.61	27	-2389.92	25	-2389.60	27	-2389.78	26	0.35	0.84	1.00	0.65	0.72	1.00	0.36	0.83	1.00
ndhC	0.11	-711.31	25	-710.46	27	-712.68	25	-710.15	27	-711.31	26	1.69	0.43	1.00	5.06	0.08	0.34	2.32	0.31	1.00
ndhD	0.22	-3958.77	25	-3958.48	27	-3959.68	25	-3958.43	27	-3958.77	26	0.59	0.75	1.00	2.50	0.29	0.92	0.69	0.71	1.00
ndhE	0.11	-635.99	25	-635.99	27	-635.53	25	-635.53	27	-635.53	26	0.00	1.00	1.00	0.00	1.00	1.00	0.00	1.00	1.00
**ndhF**	0.26	-7264.81	25	-7228.47	27	-7265.20	25	-7224.43	27	-7262.09	26	72.68	0.00	**0.00**	81.55	0.00	**0.00**	75.33	0.00	**0.00**
ndhG	0.22	-1239.29	25	-1239.29	27	-1239.31	25	-1239.22	27	-1239.26	26	0.00	1.00	1.00	0.17	0.92	1.00	0.07	0.96	1.00
ndhH	0.12	-2759.59	25	-2759.54	27	-2760.94	25	-2759.55	27	-2759.60	26	0.09	0.96	1.00	2.78	0.25	0.83	0.10	0.95	1.00
ndhI	0.10	-1215.19	25	-1215.19	27	-1215.09	25	-1214.38	27	-1214.82	26	0.00	1.00	1.00	1.43	0.49	1.00	0.89	0.64	1.00
ndhJ	0.13	-1113.51	25	-1113.51	27	-1113.38	25	-1113.38	27	-1113.38	26	0.00	1.00	1.00	0.00	1.00	1.00	0.00	1.00	1.00
ndhK	0.25	-1576.85	25	-1575.39	27	-1576.96	25	-1575.40	27	-1577.23	26	2.92	0.23	1.00	3.12	0.21	0.81	3.67	0.16	0.82
petA	0.11	-2246.16	25	-2246.16	27	-2245.30	25	-2253.03	27	-2245.30	26	0.00	1.00	1.00	-15.46	1.00	1.00	-15.46	1.00	1.00

petB	0.01	-1261.55	25	-1261.54	27	-1261.55	25	-1261.56	27	-1261.55	26	0.00	1.00	1.00	-0.02	1.00	1.00	-0.02	1.00	1.00
**petD**	0.08	-964.79	25	-963.68	27	-968.68	25	-963.70	27	-964.79	26	2.22	0.33	1.00	9.94	0.01	**0.05**	2.17	0.34	1.00
petG	0.05	-222.82	25	-222.82	27	-223.79	25	-222.33	27	-222.33	26	0.00	1.00	1.00	2.91	0.23	0.83	0.00	1.00	1.00
petL	0.25	-199.26	25	-199.26	27	-199.27	25	-199.27	27	-199.27	26	0.00	1.00	1.00	0.00	1.00	1.00	0.00	1.00	1.00
petN	0.00	-137.99	25	-137.99	27	-137.99	25	-137.99	27	-137.99	26	0.00	1.00	1.00	0.00	1.00	1.00	0.00	1.00	1.00
psaA	0.06	-4330.07	25	-4330.07	27	-4332.01	25	-4330.05	27	-4330.05	26	0.00	1.00	1.00	3.92	0.14	0.57	0.00	1.00	1.00
**psaB**	0.04	-4349.55	25	-4348.24	27	-4357.64	25	-4348.28	27	-4349.58	26	2.63	0.27	1.00	18.72	0.00	**0.00**	2.61	0.27	1.00
psaC	0.00	-519.35	25	-519.35	27	-519.35	25	-519.35	27	-519.35	26	0.00	1.00	1.00	0.00	1.00	1.00	0.00	1.00	1.00
psaI	1.03	-235.59	25	-232.07	27	-235.69	25	-232.07	27	-235.59	26	7.04	0.03	0.29	7.25	0.03	0.14	7.04	0.03	0.23
psaJ	0.28	-315.31	25	-315.31	27	-315.31	25	-315.31	27	-315.31	26	0.00	1.00	1.00	0.00	1.00	1.00	0.00	1.00	1.00
psbA	0.02	-2035.98	25	-2035.98	27	-2036.68	25	-2035.97	27	-2035.97	26	0.00	1.00	1.00	1.42	0.49	1.00	0.00	1.00	1.00
psbB	0.09	-3230.47	25	-3230.47	27	-3230.41	25	-3230.41	27	-3230.41	26	0.00	1.00	1.00	0.00	1.00	1.00	0.00	1.00	1.00

psbC	0.05	-2776.21	25	-2776.19	27	-2776.56	25	-2776.19	27	-2776.21	26	0.03	0.99	1.00	0.73	0.69	1.00	0.03	0.98	1.00
psbD	0.01	-1894.19	25	-1894.19	27	-1894.21	25	-1894.21	27	-1894.19	26	0.00	1.00	1.00	0.00	1.00	1.00	-0.03	1.00	1.00
psbE	0.02	-422.18	25	-422.18	27	-422.18	25	-422.18	27	-422.18	26	0.00	1.00	1.00	0.00	1.00	1.00	0.00	1.00	1.00
psbF	0.14	-254.51	25	-254.51	27	-254.51	25	-254.51	27	-254.51	26	0.00	1.00	1.00	0.00	1.00	1.00	0.00	1.00	1.00
psbH	0.43	-539.75	25	-539.23	27	-540.34	25	-539.23	27	-539.75	26	1.04	0.59	1.00	2.22	0.33	1.00	1.04	0.59	1.00
psbI	0.14	-256.65	25	-256.65	27	-256.66	25	-256.66	27	-256.66	26	0.00	1.00	1.00	0.00	1.00	1.00	0.00	1.00	1.00
psbJ	0.18	-222.25	25	-222.23	27	-222.34	25	-222.23	27	-222.25	26	0.03	0.99	1.00	0.22	0.90	1.00	0.03	0.99	1.00
psbK	0.17	-421.83	25	-421.66	27	-422.17	25	-421.66	27	-421.84	26	0.35	0.84	1.00	1.03	0.60	1.00	0.36	0.84	1.00
psbL	0.00	-173.27	25	-173.27	27	-173.28	25	-173.27	27	-173.27	26	0.00	1.00	1.00	0.02	0.99	1.00	0.00	1.00	1.00
psbM	0.06	-177.87	25	-177.87	27	-177.88	25	-177.88	27	-177.88	26	0.00	1.00	1.00	0.00	1.00	1.00	0.00	1.00	1.00
psbN	0.05	-259.86	25	-259.85	27	-260.06	25	-259.86	27	-259.86	26	0.00	1.00	1.00	0.40	0.82	1.00	0.00	1.00	1.00
psbT	0.29	-189.64	25	-189.55	27	-189.64	25	-189.55	27	-189.64	26	0.18	0.92	1.00	0.18	0.92	1.00	0.18	0.92	1.00
psbZ	0.08	-350.35	25	-350.35	27	-350.36	25	-350.36	27	-350.36	26	0.00	1.00	1.00	0.00	1.00	1.00	0.00	1.00	1.00

**rbcL**	0.11	-3030.71	25	-3026.14	27	-3046.24	25	-3026.72	27	-3030.71	26	9.15	0.01	0.11	39.05	0.00	**0.00**	7.99	0.02	0.18
rpl14	0.06	-839.41	25	-839.41	27	-839.11	25	-839.11	27	-839.11	26	0.00	1.00	1.00	0.00	1.00	1.00	0.00	1.00	1.00
rpl16	0.18	-915.98	25	-915.98	27	-915.96	25	-915.96	27	-915.96	26	0.00	1.00	1.00	0.00	1.00	1.00	0.00	1.00	1.00
rpl20	0.20	-901.08	25	-900.94	27	-901.17	25	-900.84	27	-901.08	26	0.27	0.87	1.00	0.67	0.72	1.00	0.48	0.78	1.00
rpl22	0.31	-1323.99	25	-1323.99	27	-1323.89	25	-1323.76	27	-1324.06	26	0.00	1.00	1.00	0.24	0.88	1.00	0.59	0.74	1.00
rpl23	0.52	-463.85	25	-463.80	27	-463.85	25	-463.80	27	-463.85	26	0.09	0.95	1.00	0.10	0.95	1.00	0.09	0.95	1.00
rpl2	0.22	-1261.57	25	-1261.48	27	-1261.63	25	-1261.48	27	-1261.57	26	0.17	0.92	1.00	0.30	0.86	1.00	0.17	0.92	1.00
**rpl32**	0.15	-539.64	25	-536.69	27	-540.63	25	-535.81	27	-539.45	26	5.91	0.05	0.45	9.65	0.01	**0.05**	7.29	0.03	0.22
rpl33	0.46	-525.22	25	-524.61	27	-525.38	25	-524.59	27	-525.22	26	1.22	0.54	1.00	1.58	0.45	1.00	1.26	0.53	1.00
rpl36	0.12	-214.55	25	-214.12	27	-215.06	25	-214.27	27	-214.55	26	0.87	0.65	1.00	1.58	0.45	1.00	0.57	0.75	1.00
rpoA	0.30	-2667.83	25	-2667.74	27	-2668.15	25	-2667.61	27	-2667.83	26	0.17	0.92	1.00	1.09	0.58	1.00	0.44	0.80	1.00
**rpoB**	0.17	-7098.23	25	-7095.74	27	-7101.10	25	-7095.75	27	-7098.28	26	4.97	0.08	0.53	10.71	0.00	**0.04**	5.05	0.08	0.47

rpoC1	0.18	-4519.04	25	-4516.91	27	-4521.29	25	-4516.76	27	-4519.05	26	4.24	0.12	0.71	9.06	0.01	0.06	4.58	0.10	0.56
**rpoC2**	0.30	-10606.31	25	-10599.94	27	-10608.24	25	-10600.35	27	-10606.34	26	12.75	0.00	**0.03**	15.79	0.00	**0.00**	11.99	0.00	**0.05**
rps11	0.24	-938.01	25	-938.01	27	-937.56	25	-937.56	27	-937.56	26	0.00	1.00	1.00	0.00	1.00	1.00	0.00	1.00	1.00
rps12	0.48	-566.04	25	-566.04	27	-566.04	25	-566.05	27	-566.04	26	0.00	1.00	1.00	0.00	1.00	1.00	0.00	1.00	1.00
rps14	0.25	-692.80	25	-692.66	27	-692.82	25	-692.67	27	-692.89	26	0.27	0.87	1.00	0.30	0.86	1.00	0.45	0.80	1.00
rps15	0.39	-719.43	25	-719.10	27	-719.82	25	-719.11	27	-719.44	26	0.67	0.72	1.00	1.43	0.49	1.00	0.67	0.72	1.00
rps16	0.31	-688.38	25	-685.67	27	-688.69	25	-685.81	27	-688.38	26	5.41	0.07	0.47	5.76	0.06	0.25	5.16	0.08	0.47
rps18	0.20	-746.90	25	-746.30	27	-746.91	25	-746.18	27	-746.93	26	1.21	0.55	1.00	1.47	0.48	1.00	1.50	0.47	1.00
rps19	0.20	-534.97	25	-534.97	27	-534.98	25	-534.98	27	-534.98	26	0.00	1.00	1.00	0.00	1.00	1.00	0.00	1.00	1.00
rps3	0.16	-1476.17	25	-1476.15	27	-1476.58	25	-1476.16	27	-1476.18	26	0.03	0.98	1.00	0.84	0.66	1.00	0.04	0.98	1.00
rps4	0.25	-1300.69	25	-1300.69	27	-1300.48	25	-1300.46	27	-1300.46	26	0.00	1.00	1.00	0.05	0.98	1.00	0.00	1.00	1.00
rps7	0.07	-638.79	25	-638.79	27	-638.79	25	-638.82	27	-638.82	26	0.00	1.00	1.00	-0.05	1.00	1.00	0.00	1.00	1.00
rps8	0.19	-1022.27	25	-1022.27	27	-1021.87	25	-1021.87	27	-1021.87	26	0.00	1.00	1.00	0.00	1.00	1.00	0.00	1.00	1.00

**ycf1**	0.67	-20357.34	25	-20293.68	27	-20361.47	25	-20295.16	27	-20357.50	26	127.33	0.00	**0.00**	132.63	0.00	**0.00**	124.69	0.00	**0.00**
**ycf2**	0.78	-11906.36	25	-11888.09	27	-11907.54	25	-11887.62	27	-11906.35	26	36.53	0.00	**0.00**	39.84	0.00	**0.00**	37.47	0.00	**0.00**
ycf3	0.11	-979.20	25	-979.20	27	-979.29	25	-979.19	27	-979.19	26	0.00	1.00	1.00	0.20	0.91	1.00	0.00	1.00	1.00
ycf4	0.27	-1470.97	25	-1470.97	27	-1470.32	25	-1470.32	27	-1470.32	26	0.00	1.00	1.00	0.00	1.00	1.00	0.00	1.00	1.00

Note LH: Likelihood (likelihood); NP: Number of parameters; FDR: False Discovery Rate; P: p-value of LTR; Genes in bold had both FDR and P-value less than 0.05.
